# DNA metabarcoding unveils authenticity and adulteration in commercial Chinese polyherbal preparations: Renshen Jianpi Wan as a critical case study

**DOI:** 10.3389/fphar.2025.1584065

**Published:** 2025-04-28

**Authors:** Shilin Zhou, Ting Zhou, Yanmei Zhang, Bingqian Yang, Junmei Niu, Weixian Li, Yiyi Weitu, Faming Long, Zhenwen Liu, Jing Zhou

**Affiliations:** ^1^ School of Pharmaceutical Science and Yunnan Key Laboratory of Pharmacology for Natural Products, Kunming Medical University, Kunming, China; ^2^ Yunnan Academy of Forestry and Grassland, Kunming, China; ^3^ Gaoligong Mountain, Forest Ecosystem, Observation and Research Station of Yunnan Province, Kunming, China; ^4^ Yunnan Key Laboratory of Biodiversity of Gaoligong Mountain, Kunming, China; ^5^ College of Modern Biomedical Industry, Kunming Medical University, Kunming, Yunnan, China

**Keywords:** commercial Chinese polyherbal preparations, DNA metabarcoding, ITS2, PSBA-TRNH, quality control

## Abstract

**Objectives:**

Ensuring quality and authenticity of traditional medicines is crucial, particularly for multi-ingredient formulations like commercial Chinese polyherbal preparations (CCPPs). This study aims to authenticate Renshen Jianpi Wan (RSJPW), a classical CCPP composed of 11 prescribed botanical drugs, using DNA metabarcoding to overcome challenges in species-level identification of processed biological ingredients.

**Methods:**

We analyzed 56 commercial RSJPW products from different manufacturers and production batches, alongside eight laboratory-prepared reference samples serving as authentic controls. A dual-marker protocol combining ITS2 and psbA-trnH regions was employed, with optimized DNA extraction and PCR protocols to mitigate degradation issues.

**Results:**

Detection rates varied across samples, with the highest detection being 10 out of 11 prescribed ingredients in a single sample. The key fungal ingredient *Poria cocos* (茯苓) was consistently undetectable, likely due to DNA degradation during processing and challenges in extracting fungal DNA from complex matrices. Multiple high-abundance non-prescribed species from Fabaceae, Apiaceae, Brassicaceae, and other families were frequently detected as potential contaminants.

**Conclusions:**

This study establishes a systematic framework for molecular authentication of complex herbal formulations, providing technical support for reliable identification of botanical drugs. While DNA metabarcoding offers valuable insights into CCPP composition, authentication of heavily processed ingredients remains a significant technical limitation. The integration with complementary analytical methods such as metabolomics could provide more comprehensive quality assessment in future studies, demonstrating the necessity of multi-analytical approaches in ensuring the authenticity of traditional medicine.

## 1 Introduction

Traditional herbal medicine has been a cornerstone of global healthcare systems for millennia, offering natural therapeutic approaches deeply rooted in diverse cultural and theoretical frameworks. The COVID-19 pandemic has renewed global interest in these remedies, particularly for their potential roles in symptom management and immune system modulation, highlighting their adaptability and accessibility during public health crises (Lyu et al., 2021). This resurgence of interest coincides with the substantial growth of traditional Chinese medicine (TCM) market, which was valued at $231.3 billion in 2023 and is projected to reach $420.7 billion by 2032, with a compound annual growth rate (CAGR) of 6.87% (Business Research Insights, 2024). This expansion is driven by increasing consumer preference for natural healthcare products, greater awareness of alternative medicine, and a broader shift toward preventive health strategies (Emergen Research, 2024). Despite this market growth, significant challenges persist regarding the safety, efficacy, and authentication of traditional herbal products. These concerns are particularly acute in regions where herbal medicines serve as primary healthcare options, such as China, Bangladesh, India, Vietnam, and South Africa (Zhang B. et al., 2022). The lack of standardized regulation and comprehensive scientific validation of potential adverse effects continues to impede their global acceptance and integration into modern healthcare systems (You et al., 2022).

China, as the largest producer and consumer of herbal medicines, faces particular challenges in the authentication of Commercial Chinese Polyherbal Preparations (CCPPs) (Xia et al., 2022)—standardized pharmaceutical preparations derived from traditional herbal prescriptions. The accurate authentication of medicinal material sources is particularly critical for quality control, as the therapeutic efficacy of CCPPs directly depends on using the correct plant species in their preparation. Recent incidents have highlighted critical issues in product safety and authenticity. Documented cases include the substitution of *Isotrema manshuriensis* (Kom.) H. Huber with *Akebia quinata* (Houtt.) Decne. in Longdan Xiegan Wan (Xin et al., 2018a), or the detection of undeclared toxic aconite in Bisset (1981). Additionally, products such as Simotang (Yi et al., 2012) have raised public health concerns due to the presence of potentially carcinogenic substances like betel nut. These incidents not only jeopardize consumer trust but also reveal systemic vulnerabilities in authentication methods.

Traditional authentication methods, including microscopic identification (Zhao et al., 2005) and thin-layer chromatography (TLC) (Zhang et al., 2018), have long been employed in the quality control of botanical drugs. However, these methods face significant limitations in dealing with the complexity of CCPPs, which often involve processed and multi-ingredient formulations. As herbal formulations become more sophisticated and diverse, there is a growing need for more advanced approaches to ensure product quality and regulatory compliance. DNA metabarcoding, introduced by Taberlet et al. (2012) in 2012, has emerged as a transformative tool for species identification by enabling the simultaneous detection of multiple species within complex samples. This method uses genetic markers to amplify specific regions of DNA, allowing for the comprehensive analysis of species composition in herbal preparations. Coghlan et al. (2012) utilized high-throughput sequencing to analyze herbal preparations and identified a wide range of plant and animal species, including toxic and endangered species. Since then, DNA metabarcoding has been increasingly adopted for biodiversity biomonitoring and environmental assessments (Miya, 2022; Pawlowski et al., 2022), and its application in CCPP authentication has gained increasing recognition for its accuracy and efficiency (Arulandhu et al., 2017; Gao et al., 2019; Liu et al., 2019; Seethapathy et al., 2019; Yu et al., 2021; Shah et al., 2023).

This study focuses on Renshen Jianpi Wan (RSJPW), a representative traditional CCPP with extensive clinical application. The formula, first recorded in *Zhengzhi Zhunsheng Leifang* by Kentang Wang during Ming Dynasty, includes 11 botanical drugs with distinct therapeutic roles. These botanical drugs, including Ginseng Radix et Rhizoma (Renshen), Atractylodis Macrocephalae Rhizoma (Baizhu), Poria (Fuling), and Dioscoreae Rhizoma (Shanyao) *etc*., are commonly used to strengthen the spleen and stomach, promote digestion, regulate qi, and alleviate various gastrointestinal disorders (Zu et al., 2023). Its widespread use is evidenced by its current market presence. According to the National Medical Products Administration database, 141 pharmaceutical companies hold production licenses for RSJPW, with over 40 manufacturers actively selling their products. However, quality concerns have emerged alongside its increasing market demand. For instance, the National Medical Products Administration reported quality deficiencies in five batches of RSJPW from three manufacturers in 2010 (The Central People’s Government of the People’s Republic of China, 2010). Current quality control methods, as specified in the Chinese Pharmacopoeia Committee (2020), only provide authentication protocols for 6 out of the 11 prescribed botanical drugs and rely primarily on operator-dependent techniques. Moreover, these methods cannot effectively detect unauthorized substitutions or adulterations. The complex manufacturing process of RSJPW, which involves pulverization, drying, and high-temperature processing, poses additional challenges for quality control by potentially degrading DNA and other chemical markers. These factors make RSJPW an ideal candidate for exploring advanced authentication approaches.

In this study, we developed and validated a DNA metabarcoding-based method for systematic authentication of the biological composition of RSJPW. By analyzing both reference materials and commercial samples, we aimed to establish a reliable authentication strategy that addresses the technical challenges in authenticating complex herbal formulations. Our method was specifically designed to detect both legitimate botanical drugs and potential adulterants, with particular attention to discriminating between *Panax ginseng* C. A. Mey. and its common adulterant (Chen et al., 2013), *Panax quinquefolius* L. This research provides not only a practical tool for RSJPW species authentication but also demonstrates a systematic approach to DNA-based authentication of CCPPs.

## 2 Materials and methods

### 2.1 Materials

#### 2.1.1 Collection and identification of raw materials

The RSJPW formula comprises 11 medicinal ingredients (Table 1): Ginseng Radix et Rhizoma (Renshen), Atractylodis Macrocephalae Rhizoma (Baizhu, stir-fried with wheat bran), Poria (Fuling), Dioscoreae Rhizoma (Shanyao), Citri Reticulatae Pericarpium (Chenpi), Aucklandiae Radix (Muxiang), Amomi Fructus (Sharen), Astragali Radix (Huangqi, honey-processed), Angelicae Sinensis Radix (Danggui), Ziziphi Spinosae Semen (Suanzaoren, stir-fried), and Polygalae Radix (Yuanzhi, processed). Raw materials were collected from authorized traditional Chinese medicine pharmacies and certified online pharmaceutical platforms during 2022–2023. Additionally, Panacis Quinquefolii Radix (*P. quinquefolius*, Xiyangshen), a common adulterant of Ginseng Radix et Rhizoma, was included as a positive control. All materials were stored in airtight bags at room temperature (20–25°C) with relative humidity maintained below 60% until analysis.

**TABLE 1:** Composition and Botanical Sources of prescribed ingredients in RSJPW (Chinese Pharmacopoeia, 2020).

Herbal ingredients	Dosage (g)	Ratio (%)	Medicinal part	Botanical source	Family
Ginseng Radix et Rhizoma (Renshen)	25	4	Root and Rhizome	*Panax ginseng* C.A. Mey	Araliaceae
Atractylodis Macrocephalae Rhizoma (Baizhu)	150	24	Rhizome	*Atractylodes macrocephala* Koidz	Asteraceae
Poria (Fuling)	50	8	Sclerotium	*Poria cocos* (Schw.) Wolf	Polyporaceae
Dioscoreae Rhizoma (Shanyao)	100	16	Rhizome	*Dioscorea polystachya* Thunb	Dioscoreaceae
Citri Reticulatae Pericarpium (Chenpi)	50	8	Fruit	*Citrus reticulata* Blanco	Rutaceae
Aucklandiae Radix (Muxiang)	12.5	2	Root	*Aucklandia costus* Decne	Asteraceae
Amomi Fructus (Sharen)	25	4	Fruit	*Amomum villosum* Lour	Zingiberaceae
				*A. villosum* var. *Xanthioides* T.L. Wu et Senjen	
				*A. longiligulare* T.L. Wu	
Astragali Radix (Huangqi)	100	16	Root	*Astragalus membranaceus* var. *Mongholicus* (Bye.) Hsiao	Fabaceae
				*A. membranaceus* (Fisch.) Bge	
Angelicae Sinensis Radix (Danggui)	50	8	Root	*Angelica sinensis* (Oliv.) Diels	Apiaceae
Ziziphi Spinosae Semen (Suanzaoren)	50	8	Seed	*Ziziphus jujuba* var. *Spinosa*(Bunge) Hu ex.H.F. Chou	Rhamnaceae
Polygalae Radix (Yuanzhi)	25	4	Root	*Polygala tenuifolia* Willd	Polygalaceae
				*P*. *sibirica* L.	
**Panacis Quinquefolii Radix (Xiyangshen)**	12.5	2	Root	*Panax quinquefolius* L.	Araliaceae

The morphological identification of botanical drugs was performed by Dr. Jing Zhou according to the macroscopical identification criteria specified in the Chinese Pharmacopoeia (2020), including examination of characteristic features such as shape, size, color, surface, texture, fracture (cross-sectional appearance), odor, and other distinctive physical properties of the medicinal parts. For molecular identification, DNA was extracted from each botanical drug separately. The internal transcribed spacer 2 (ITS2) and *psb*A-*trn*H intergenic spacer regions were amplified following the guidelines in the Chinese Pharmacopoeia Committee (2020). However, as Poria (Fuling), a fungal medicinal material, cannot be amplified using the ITS2 and *psb*A-*trn*H primers specified in the Pharmacopoeia, we used ITS primers ITS1 (5′-TCC​GTA​GGT​GAA​CCT​GCG​G-3′) and ITS4 (5′-TCC​GCT​TAT​TGA​TAT​GC-3′) for its identification (Qin et al., 2023). The sequencing data were analyzed using BLAST against the GenBank database (NCBI), with a sequence similarity threshold of ≥99% and query coverage ≥95% for species-level identification. This molecular approach, combined with morphological identification, ensured the accurate identification of the collected raw materials for subsequent quality analysis.

#### 2.1.2 Collection of commercial samples

A total of 56 commercial RSJPW samples from 12 manufacturers were collected in 2022–2023. Each manufacturer contributed 2 to 6 batches of samples, which were purchased from major online pharmaceutical platforms and licensed brick-and-mortar pharmacies across China (Supplementary Table S1). All samples were within their shelf life and stored according to the manufacturer’s instructions until analysis.

#### 2.1.3 Preparation of reference materials

Two sets of reference materials were prepared to validate the detection method. The authenticated raw materials (including *P. quinquefolius*) were individually ground and sieved. For the standard formula reference set (RF05−RF08), the 11 medicinal ingredients were weighed and mixed according to the proportions specified in the Chinese Pharmacopoeia (2020) (Table 1). The mixture was then processed into pills (10 g each) according to standard procedures. For the positive control set (RF01−RF04), *P. quinquefolius* powder was incorporated at 1.96% w/w (equivalent to the proportion of Aucklandiae Radix) into the standard formula before pill formation.

### 2.2 Development of DNA metabarcoding method for authentication

#### 2.2.1 DNA extraction

After preliminary comparison of two extraction methods (CTAB and commercial kit), the plant genomic DNA extraction kit (Beijing Biomed Gene Technologies Co., Ltd.) was selected and optimized for CCPPs. The protocol was optimized through the following modifications: (1) cleaning samples with 75% ethanol; (2) adding Tris-HCl buffer (pH 8.0) during grinding; and (3) extending the water bath duration. To maximize DNA recovery, extracts were processed in triplicate with subsequent combination during AC column adsorption.

DNA quality and concentration were assessed using a NanoDrop 2000 spectrophotometer (Thermo Fisher Scientific, United States).

#### 2.2.2 PCR amplification

ITS2 and *psb*A-*trn*H regions were selected as complementary markers following recommendations from the Consortium for the Barcode of Life (CBOL) and previous studies demonstrating their effectiveness in botanical drug authentication (Kress and Erickson, 2007; Chen et al., 2010; Yao et al., 2022). ITS2 provides superior species-level resolution for medicinal plants, and *psb*A-*trn*H offers robust discriminatory power even in processed materials. This dual-marker approach has been validated in multiple studies as particularly effective for traditional medicine authentication, providing complementary identification power when analyzing complex botanical formulations (Arulandhu et al., 2017). Universal primers were modified with sample-specific 6-bp tags at their 5′ ends (Supplementary Tables S1, S2). PCR was performed using TransStart Fastpfu DNA Polymerase (TransGen AP221-02) in a 20 μL reaction system: 10 μL of 2× Pro Taq buffer, 0.8 μL of forward primer (5 μM), 0.8 μL of reverse primer (5 μM), 4 μL of template DNA (10 ng/μL), and ddH_2_O added to a final volume of 20 μL. Specific amplification conditions for each marker are detailed in Supplementary Table S2.

#### 2.2.3 Library construction and sequencing

The PCR products were visualized on 2% agarose gel electrophoresis and purified using the AxyPrep DNA Gel Extraction Kit (AXYGEN). DNA quantification was performed using the QuantiFluor™-ST Blue Fluorescence Quantification System (Promega). Libraries were constructed using the TruSeq™ DNA Sample Prep Kit according to the manufacturer’s protocol. Paired-end 300 bp sequencing was performed on the Illumina MiSeq platform by Majorbio Bio-pharm Technology Co., Ltd. (Shanghai, China). The raw sequence data has been submitted to NCBI wth the accession number of PRJNA1242227.

#### 2.2.4 Bioinformatic analysis

Raw sequence data processing and analysis were performed using QIIME two software v. 2021.2 (Hall and Beiko, 2018). After quality assessment using q2-demux, primers and sample-specific tags were removed with q2-cutadapt. Quality filtering parameters were set as follows: minimum quality score of 25, trimming of first 10 bases, and maximum expected errors of 2.0. The filtered sequences were processed using q2-dada2 for denoising, paired-end read merging and chimera removal. Feature tables and representative sequences were generated, and their statistics were analyzed using the feature-table summarize command. To minimize potential sequencing artifacts and improve data reliability, Amplicon Sequence Variants (ASVs) with fewer than 10 reads per sample were filtered out, a threshold determined based on the complexity of botanical drug matrices and sequencing depth.

For accurate species identification, each representative sequence was manually compared against the NCBI GenBank database using BLAST, with stringent criteria optimized for RSJPW authentication (sequence similarity ≥98% and query coverage ≥95%). To ensure specificity in the context of traditional Chinese medicine, only plant species documented in Chinese pharmacopoeia were considered as valid matches to avoid false positives from closely related species. For diversity analysis, alpha diversity (Shannon index) was calculated using the q2-diversity plugin to evaluate species richness and evenness across samples. Beta diversity analysis was performed using Bray-Curtis distances, and the results were visualized through Principal Coordinates Analysis (PCoA) to examine compositional differences between samples. The analysis was optimized to address the unique challenges of RSJPW, focusing on comparing species composition and relative abundances across different commercial samples to evaluate formula consistency and detect potential adulterations in this complex preparation.

#### 2.2.5 Method validation

The reproducibility and reliability of the established workflow was validated through parallel reference samples. Two sets of quadruplicate reference samples were prepared: RF01−RF04 (spiked with *P. quinquefolius*) and RF05−RF08 (without *P. quinquefolius*) serving as positive controls and quality control materials, respectively. The validation protocol included: (1) Technical reproducibility through consistency analysis of species detection across quadruplicate samples; (2) Method specificity by comparing species profiles between samples with and without *P. quinquefolius*; (3) Cross-validation of species detection between ITS2 and *psb*A*-trn*H markers. Sixteen unique 6-bp tags were designed and appended to the 5′ end of the universal primers for both ITS2 and *psb*A-*trn*H sequences (Supplementary Table S1) to distinguish PCR amplicons from different samples.

## 3 Results

### 3.1 Authentication of RSJPW raw materials

All 11 medicinal ingredients used in the RSJPW formula, along with the positive control *P. quinquefolius*, were verified through both morphological and molecular approaches. Morphological characteristics of all materials matched their corresponding authentic descriptions in Chinese Pharmacopoeia (2020). For molecular authentication, ITS2 and *psb*A-*trn*H sequences showed ≥99% similarity to their corresponding authentic species in reference databases, except for the fungal material *Poria cocos* (Schw.) Wolf (Poria) which was authenticated using ITS region due to its taxonomic classification. All raw materials were authenticated as genuine species documented in Chinese Pharmacopoeia (2020).

### 3.2 Development and validation of DNA metabarcoding workflow for botanical ingredient authentication

**FIGURE 1:**
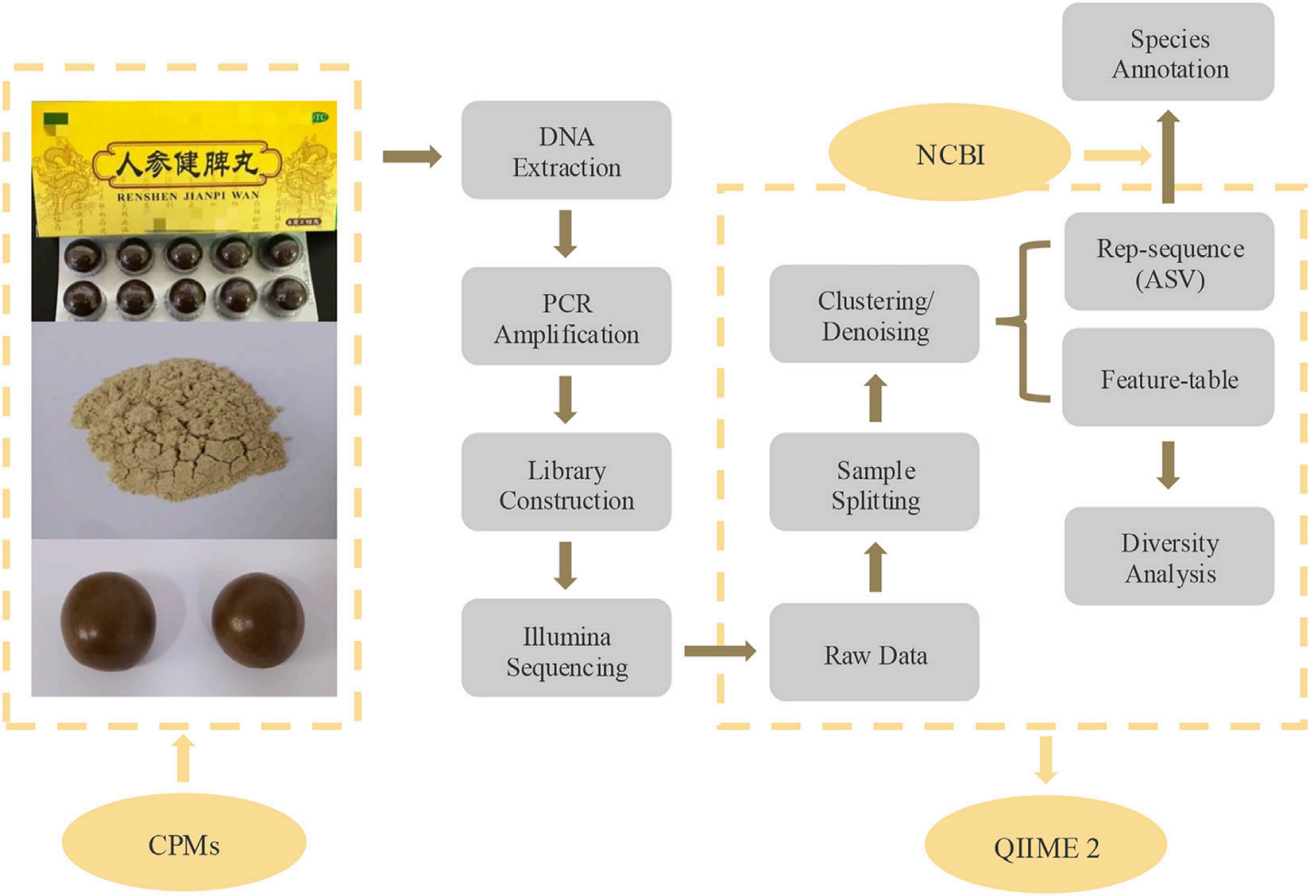
Workflow for biological ingredient monitoring of RSJPW using DNA metabarcoding.

A stepwise DNA metabarcoding workflow was established to authenticate and profile the biological ingredients of RSJPW (Figure 1), which comprises four main steps: sample processing, molecular amplification, high-throughput sequencing, and bioinformatic species annotation.

**FIGURE 2:**
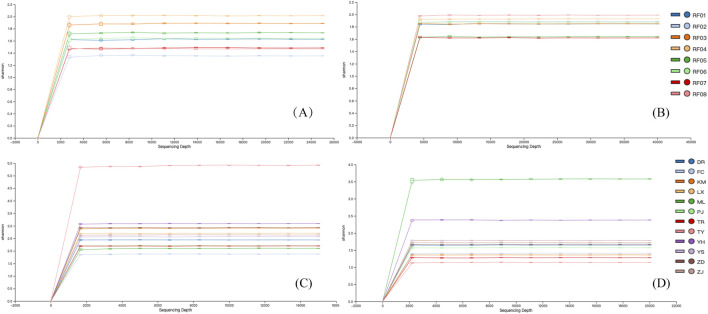
Rarefaction curves based on Shannon index. **(A)** ITS2 sequences from reference samples; **(B)** *psb*A-*trn*H sequences from reference sanples; **(C)** ITS2 sequences from commercial samples; **(D)** *psb*A-*trn*H sequences from commercial samples.

The sequencing quality assessment showed robust data generation, with a total of 291,339 and 439,257 reads obtained for ITS2 and *psb*A-*trn*H regions, respectively, all of which achieved a mean Q30 quality score exceeding 95% (Table 2). The Shannon rarefaction curves (Figures 2A, B) reached clear plateaus, indicating sufficient sequencing coverage to capture species diversity within RSJPW samples.

**TABLE 2:** Overview of sequencing metrics for reference and commercial samples.

Parameters	Marker	Amplicon size (bp)	Sequencing length (bp)	Raw reads	Total bases	Average Q30 score (%)	Clean reads	Final reads	Recovery rate (%)	Initial ASVs	Final ASVs
References samples	ITS2	500	300	291,339	131,490,894	95.73	216,384	216,008	74.27	146	66
	*psb*A-*trn*H	500	300	439,257	160,418,602	97.43	370,110	369,815	84.26	116	59
Commercial samples	ITS2	500	300	1,764,844	786,454,412	94.76	1,265,805	1,263,271	71.72	1,477	941
	*psb*A-*trn*H	500	300	1,642,373	588,937,401	96.71	1,482,386	1,480,963	90.26	1,125	842

Note: Clean reads represent the remaining reads after Qiime two software quality control; Final reads represent the number of remaining reads after ASVs, with fewer than 10 reads are removed.

**FIGURE 3:**
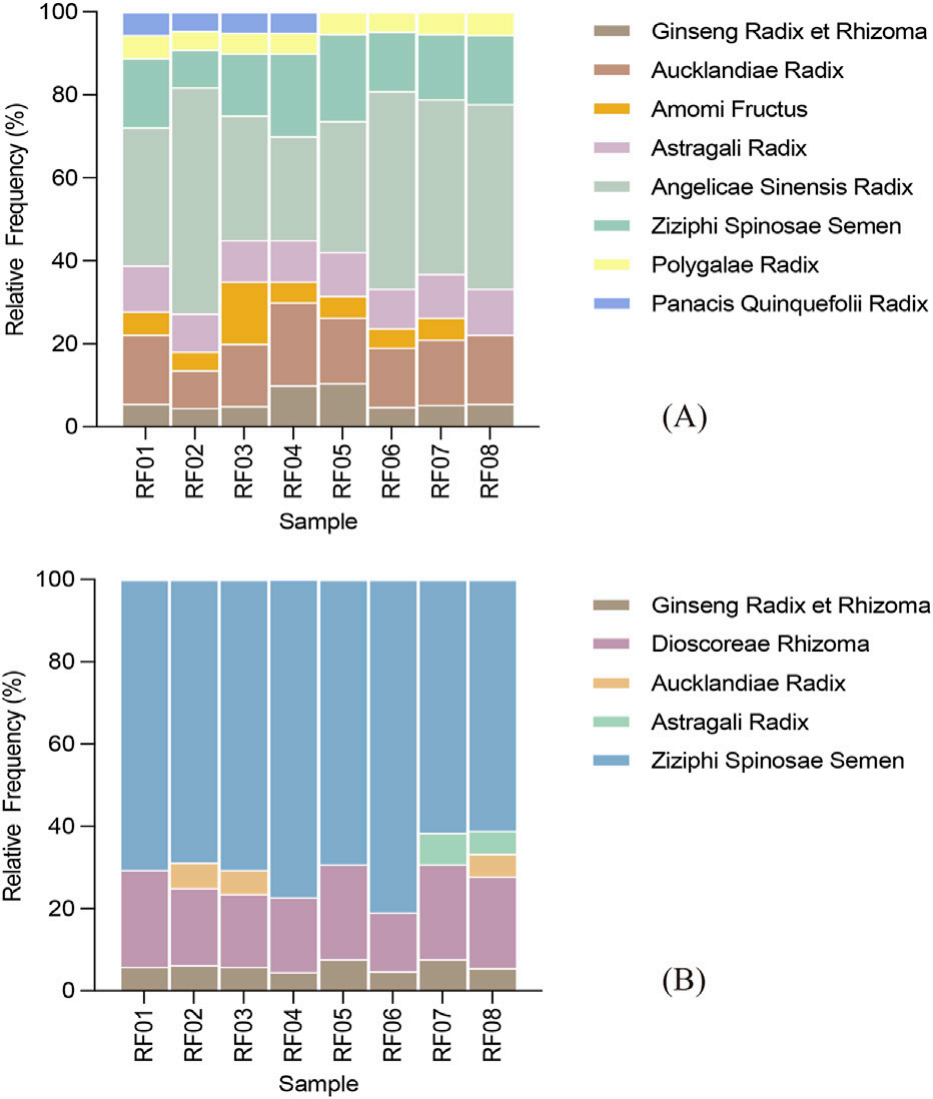
ASV abundances for detected species in reference samples. **(A)** ASV abundances through ITS2 analysis; **(B)** ASV abundances through *psb*A-*trn*H analysis.

The workflow validation was conducted using eight laboratory-prepared RSJPW reference samples, including four spiked with *P. quinquefolius* (RF01−RF04) and four standard formula samples (RF05−RF08). After quality filtering, ITS2 and *psb*A-*trn*H regions yielded 66 and 59 ASVs, respectively (Table 2; Supplementary Table S3). ITS2 marker detected seven prescribed ingredients with relatively consistent detection across replicates, including *P*. *ginseng* C. A. Mey., *Aucklandia costus* Decne., *Amomum villosum* Lour., *Astragalus membranaceus* (Fisch.) Bge., *Angelica sinensis* (Oliv.) Diels, *Z. jujuba* var*. Spinosa* (Bunge) Hu ex.H.F. Chou, and *Polygala tenuifolia* Willd. In the cross-validation analysis using *psb*A-*trn*H marker, *P. ginseng* and *Ziziphus jujuba* var. *Spinosa* were consistently detected, while *Dioscorea polystachya* Thunb. Was uniquely identified by this marker, demonstrating complementary detection capabilities of the two markers. The positive control *P. quinquefolius* was specifically identified in spiked samples (RF01−RF04) while absent in standard formula samples (RF05−RF08), demonstrating the workflow’s sensitivity and its ability to identify formula adulteration. However, despite their successful authentication in raw materials, *Citrus reticulata* Blanco, *P. cocos*, and *Atractylodes macrocephala* Koidz. Were not detected in the prepared formula samples by either marker (Table 3; Figure 3).

**TABLE 3:** Detection of prescribed ingredients in RSJPW reference samples using ITS2 and *psb*A-*trn*H markers.

Herbal ingredients	ITS2								*psb*A-*trn*H							
	RF01	RF02	RF03	RF04	RF05	RF06	RF07	RF08	RF01	RF02	RF03	RF04	RF05	RF06	RF07	RF08
Ginseng Radix et Rhizoma (Renshen)	√	√	√	√	√	√	√	√	√	√	√	√	√	√	√	√
Atractylodis Macrocephalae Rhizoma (Baizhu)	--	--	--	--	--	--	--	--	--	--	--	--	--	--	--	--
Poria (Fuling)	--	--	--	--	--	--	--	--	--	--	--	--	--	--	--	--
Dioscoreae Rhizoma (Shanyao)	--	--	--	--	--	--	--	--	√	√	√	√	√	√	√	√
Citri Reticulatae Pericarpium (Chenpi)	--	--	--	--	--	--	--	--	--	--	--	--	--	--	--	--
Aucklandiae Radix (Muxiang)	√	√	√	√	√	√	√	√	--	√	√	--	--	--	--	√
Amomi Fructus (Sharen)	√	√	√	√	√	√	√	--	--	--	--	--	--	--	--	--
Astragali Radix (Huangqi)	√	√	√	√	√	√	√	√	--	--	--	--	--	--	√	√
Angelicae Sinensis Radix (Danggui)	√	√	√	√	√	√	√	√	--	--	--	--	--	--	--	--
Ziziphi Spinosae Semen (Suanzaoren)	√	√	√	√	√	√	√	√	√	√	√	√	√	√	√	√
Polygalae Radix (Yuanzhi)	√	√	√	√	√	√	√	√	--	--	--	--	--	--	--	--
**Panacis Quinquefolii Radix (Xiyangshen)**	**√**	**√**	**√**	**√**	--	--	--	--	--	--	--	--	--	--	--	--

Note: -: the ingredient was not detected for the sample. Boldface indicates positive control herbal ingredients.

### 3.3 Analysis of commercial RSJPW samples

#### 3.3.1 Sequencing results and prescribed ingredients detection

**FIGURE 4:**
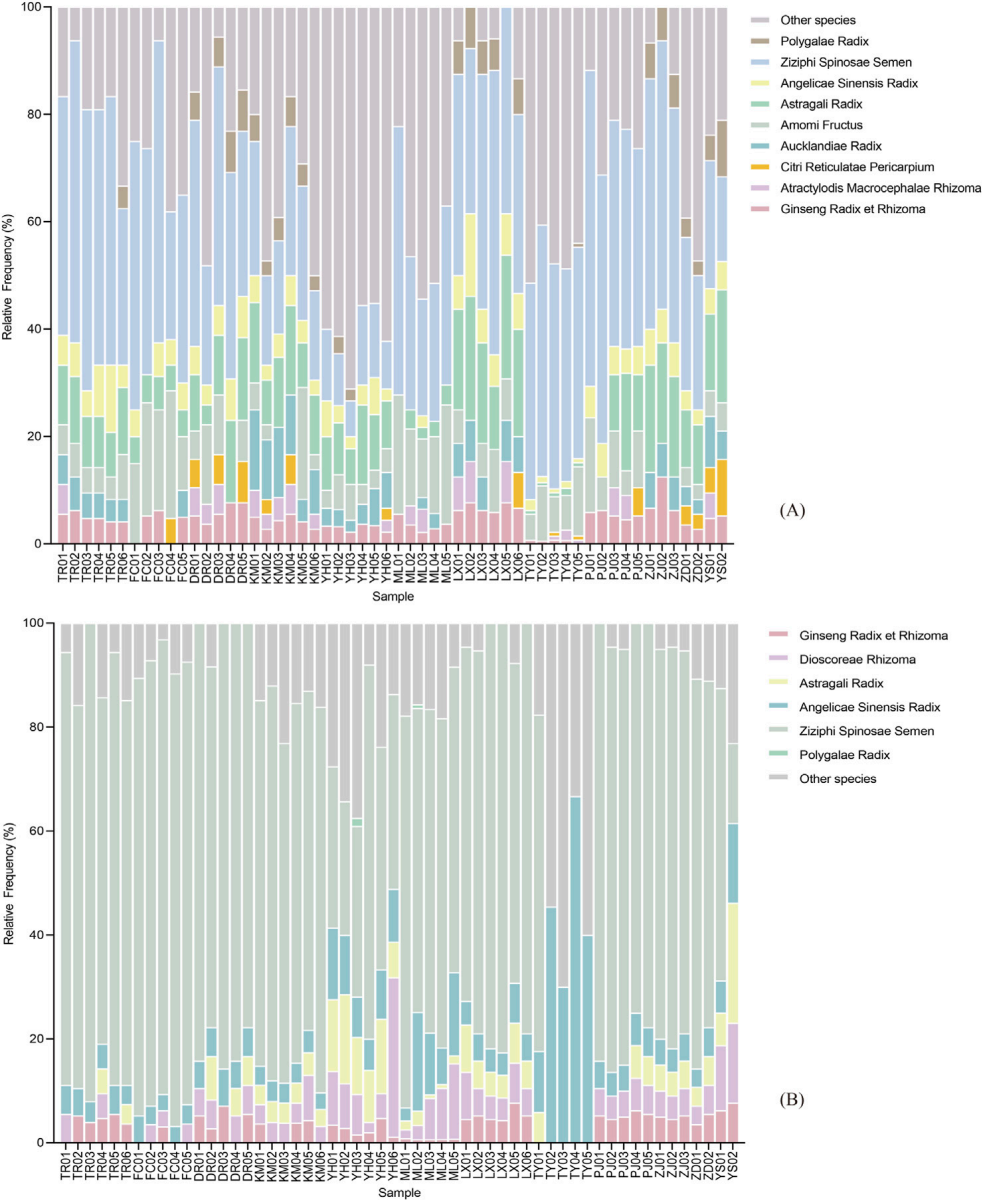
ASV Abundances for detected species across commercial RSJPW samples. **(A)** ASV abundances through ITS2 analysis; **(B)** ASV abundances through *psb*A-*trn*H analysis.

High-throughput sequencing of 56 commercial RSJPW samples using ITS2 and *psb*A-*trn*H markers yielded high-quality data (Q30 > 94%, Table 2). Shannon rarefaction curves also plateaued for all samples, indicating sufficient sequencing depth for species diversity assessment (Figures 2C, D). Post-quality filtering, ITS2 sequencing generated 941 ASVs across commercial samples (13–175 ASVs per sample), while *psb*A-*trn*H produced 842 ASVs (3–151 ASVs per sample) (Figure 4; Supplementary Tables S4, S5). These values considerably exceeded those from reference samples (66 and 59 ASVs for ITS2 and *psb*A-*trn*H, respectively), indicating enhanced biological complexity in commercial formulations.

The two markers exhibited complementary detection patterns for prescribed ingredients. ITS2 demonstrated complete detection (100%) for *Z*. *jujuba* var. *spinosa*, with high detection rates for *P*. *ginseng* and *A*. *membranaceus* (94.64%), as well as *A*. *sinensis* (91.07%). However, ITS2 failed to detect *P*. *cocos* and *D*. *polystachya* across all samples. For *psb*A-*trn*H analysis, *A. sinensis* showed complete detection (100%), followed by *Z*. *jujuba* var*. spinosa* (92.86%). Notably, *psb*A-*trn*H uniquely identified *D. polystachya* (78.57%) but showed limited detection of *P*. *tenuifolia* (3.57%). Five prescribed ingredients (*A*. *macrocephala*, *P. cocos*, *C*. *reticulata*, *A*. *costus*, and *A*. *villosum*) remained undetected by *psb*A-*trn*H. When combining both markers, 10 out of 11 prescribed ingredients were successfully detected in the commercial samples, with detection frequencies varying from 3.57% to 100%. Thus, the combined use of both markers enabled the detection of a broader range of species, with each marker contributing unique detection capabilities.

The relative abundance of detected species, inferred from sequencing reads, showed substantial variation among samples (Supplementary Tables S6, S7), suggesting potential differences in ingredient proportions across manufacturers. Due to the variable detection patterns across the 56 batches and incomplete detection of all prescribed ingredients, direct correlation analysis between read abundance and ingredient proportions in the original RSJPW formula was not feasible.

#### 3.3.2 Non-prescribed species identification

Metabarcoding analysis using dual markers (ITS2 and *psb*A-*trn*H) revealed substantial non-prescribed species contamination in commercial RSJPW samples. These species can be classified into three categories: non-formula medicinal plants, food crops, and wild plants.

A total of 120 non-prescribed species (Tables 4, 5; Supplementary Table S8) were identified using the ITS2 region, representing 43 different families. Species from the Leguminosae and Apiaceae families were the most prevalent. *Verbena officinalis* L. had the highest relative abundance with 23,911 reads across 36 ASVs, and was detected in 25% of samples. Other frequently detected species included *Alnus nepalensis* D. Don (44.64%), *Cucurbita moschata* Duchesne (39.29%), and *Triticum aestivum* L. (42.86%). Moreover, we also identified closely related species or potential substitutes of the prescribed ingredients, such as *Hedysarum polybotrys* Hand.-Mazz., a substitute for *A. membranaceus*, and several confusable varieties of *P. ginseng*, including *P. quinquefolius*, *P*. *japonicus* (T. Nees) C. A. Meyer and *Codonopsis pilosula* (Franch.) Nannf., *etc*. Beyond plant species, two fungi- *Bacillus altitudinis* and *Dimargaris bacillispora*, were also detected by the ITS2 sequence, both exclusively in samples from the manufacturer TY.

**TABLE 4:** Non-prescribed species detected in commercial RSJPW samples based on ITS2 sequences.

Family	Latin name	Reads no.	ASV no.	Sample no.	Detection frequency (%)	Possible source category
Verbenaceae	*Verbena officinalis* L.	23,911	36	14	25.00	Medicinal plant
Brassicaceae	*Brassica napus* L.	18,182	58	15	26.79	Food crop
Cucurbitaceae	*Cucurbita moschata* (Duch. Ex Lam.) Duch. Ex Poir	11,870	15	22	39.29	Food crop
Apiaceae	*Peucedanum caespitosum* H. Wolff	9,926	17	6	10.71	Medicinal plant
Brassicaceae	*Raphanus sativus* L.	5,067	9	14	25.00	Food crop
Apiaceae	*Peucedanum praeruptorum* Dunn	4,776	11	8	14.29	Medicinal plant
Asteraceae	*Artemisia argyi* H. Lév. and Vaniot	4,707	10	1	1.79	Medicinal plant
Poaceae	*Setaria viridis* (L.) P. Beauv	4,356	2	6	10.71	Wild plant
Betulaceae	*Alnus nepalensis* D. Don	4,351	10	25	44.64	Wild plant
Convolvulaceae	*Cuscuta australis* R. Br	3,977	7	17	30.36	Medicinal plant
Poaceae	*Triticum aestivum* L.	3,938	9	24	42.86	Food crop
Cucurbitaceae	*Cucumis sativus* L.	3,122	12	3	5.36	Food crop
Pinaceae	*Pinus tabuliformis* Carrière	1,438	6	12	21.43	Wild plant
Paeoniaceae	*Paeonia* × *suffruticosa* Andrews	1,040	3	3	5.36	Medicinal plant
Amaryllidaceae	*Hymenocallis littoralis* (Jacq.) Salisb	987	20	6	10.71	Wild plant

Note: This table shows the 15 species with the highest sequencing read counts. Detection frequency = (Number of samples where the species is detected/Total number of samples) × 100%.

**TABLE 5:** Related species of prescribed ingredients detected in commercial RSJPW samples based on ITS2 sequences.

Family	Latin name	Reads no.	ASV no.	Sample no.	Detection frequency (%)	Related prescribed species
Fabaceae	*Hedysarum polybotrys* Hand.-Mazz	343	3	5	8.93	*Astragalus membranaceus*
Rhamnaceae	*Ziziphus mauritiana* Lam	551	8	2	3.57	*Ziziphus jujuba*
Araliaceae	*Panax quinquefolius* L.	95	1	2	3.57	*Panax ginseng*
Araliaceae	*Panax japonicus* (T. Nees) C. A. Meyer	11	1	1	1.79	*Panax ginseng*
Campanulaceae	*Codonopsis pilosula* (Franch.) Nannf	116	1	2	3.57	*Panax ginseng*
Rutaceae	*Citrus sinensis* (L.) Osbeck	16	1	2	3.57	*Citrus reticulata*
Apiaceae	*Angelica acutiloba* (Siebold and Zucc.) Kitag	12	1	1	1.79	*Angelica sinensis*
Zingiberaceae	*Amomum compactum* Soland ex Maton	65	1	1	1.79	*Amomum villosum*

Note: Detection frequency = (Number of samples where the species is detected/Total number of samples) × 100%.

Parallel analysis using the *psb*A-*trn*H sequence verified the findings of ITS2 in terms of species composition patterns and revealed an additional 55 non-prescribed species (Supplementary Table S9) from 26 different families. Species from the Leguminosae and Salicaceae families were predominant. *Arachis hypogaea* L. had the highest relative abundance, with a total of 62,146 reads across 9 ASVs, and a detection frequency of 64.29%. In addition, *Salix alba* L. (48.21%), *Polygonum multiflorum* Thunb. (16.07%), and *Ziziphus mauritiana* Lam., a known adulterant of *Z*. *jujuba* var. *spinosa*, (16.07%) were also frequently detected. Notably, *Paeonia rockii* (S.G. Haw and Lauener) T. Hong and J.J. Li, a first-class protected plant in China, was detected in 10.71% of the samples, with a total read count of 3,135.

#### 3.3.3 Batch-to-batch and manufacturer variation

Metabarcoding analysis successfully detected all prescribed species except *P. cocos*. However, distinct inter-manufacturer and inter-batch variations in both ASV abundance and species detection profiles were revealed (Supplementary Tables S4, S5).

ITS2 sequencing demonstrated marked manufacturer-specific variation in ASV diversity. *Ziziphus jujuba* var. *spinosa* exhibited the most substantial variation, with manufacturer TY samples yielding 52–82 ASVs compared to <12 ASVs from other manufacturers. *Amomum villosum* displayed moderate variation (1–18 ASVs). In contrast, core prescribed species, including *P. ginseng*, *A. macrocephala*, *C. reticulata*, *A. costus*, *A. membranaceus*, *A. sinensis*, and *P. tenuifolia*, maintained consistent ASV profiles (1–4 ASVs) across all manufacturers. The *psb*A-*trn*H marker analysis corroborated these variation patterns, with notably high ASV counts in *Z. jujuba* var. *spinosa* samples from manufacturer ML (77–94 ASVs). Additionally, *D. polystachya* and *A. sinensis* showed elevated diversity in specific manufacturers, while the remaining prescribed species maintained relatively stable profiles across all sources.

**FIGURE 5:**
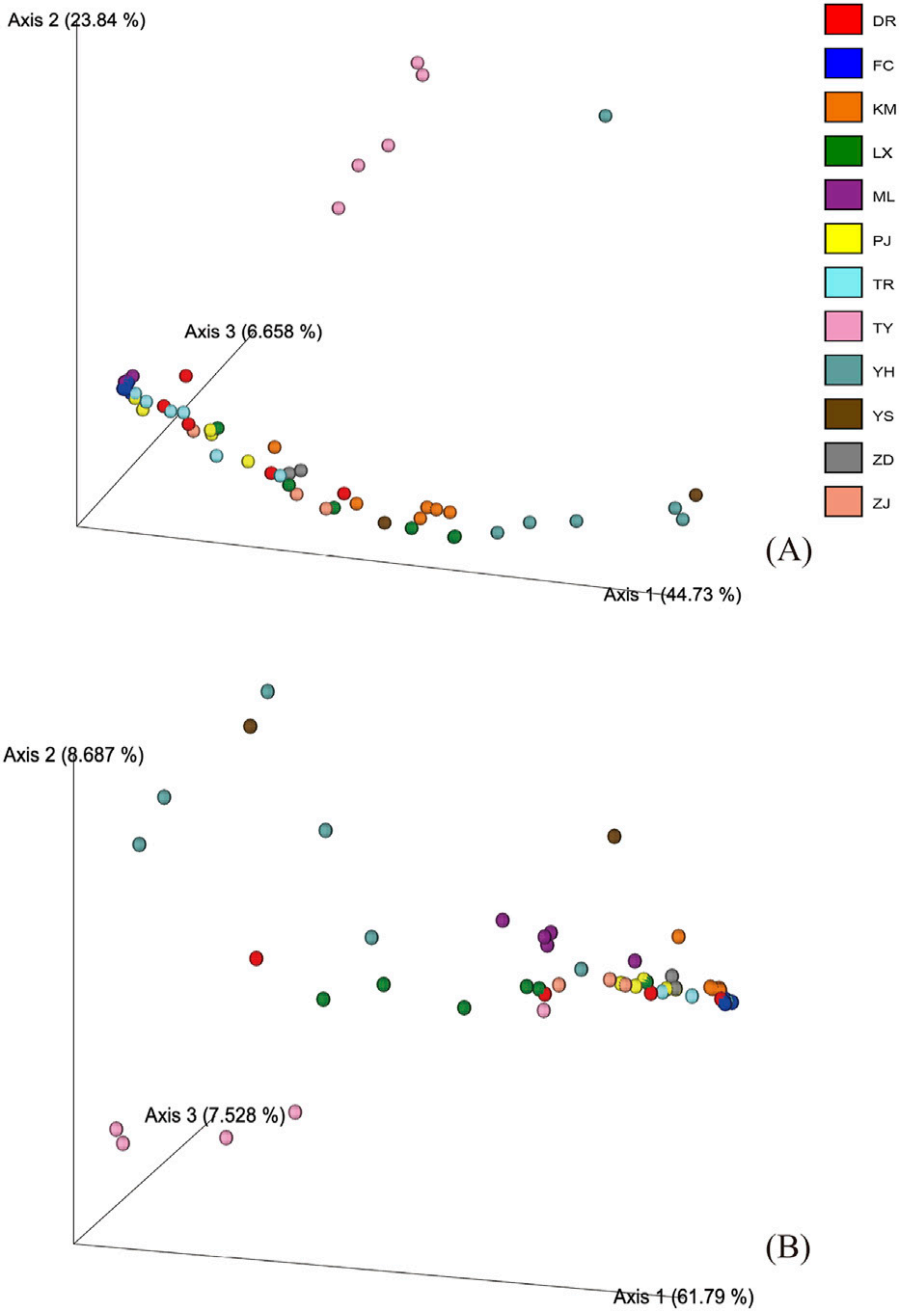
PCoA plot of species detected in commercial samples based on Bray-Curtis distance. **(A)** Analysis results of ITS2 sequences. **(B)** Analysis results of *psb*A-*trn*H sequences.

Species detection rates exhibited substantial heterogeneity across manufacturers and batches. In ITS2 analysis, manufacturers showed varying levels of consistency in species detection: YH and TR demonstrated reliable performance by consistently detecting 6 out of 11 prescribed species across all batches, while other manufacturers displayed more variable detection patterns. Detection capability ranged from high-performing batches, such as KM02 which detected up to 9 species, to notably poor performance in some cases, with certain TY batches detecting only a single species in *psb*A-*trn*H analysis. This heterogeneity in detection rates is consistent with the observed variations in ASV profiles and is further supported by PCoA analysis (Figure 5), which revealed manufacturer-specific clustering patterns ranging from highly cohesive (as seen in TY samples) to broadly dispersed distributions, suggesting varying levels of standardization and quality control among manufacturers.

## 4 Discussion

### 4.1 DNA metabarcoding for botanical drug authentication in CCPP: advantages and challenges

Commercial Chinese polyherbal preparations (CCPPs) are typically composed of multiple biological drugs with complex sources. The frequent inconsistencies between pharmacopoeia-specified materials and their substitutes present unique challenges in quality assessment, which directly impact therapeutic reliability and safety. While current analytical methods, including chromatography and mass spectrometry, are valuable for specific aspects of quality assessment, they lack the capability for simultaneous multi-ingredient authentication (Wang et al., 2021). DNA metabarcoding overcomes this limitation by enabling comprehensive detection of multiple species in complex mixtures (Taberlet et al., 2012; Urumarudappa et al., 2020; Pandit et al., 2021; Raclariu et al., 2021; Travadi et al., 2023). This approach demonstrated high efficiency in our study by successfully identifying 10 out of 11 prescribed ingredients in RSJPW samples. The complementary use of ITS2 and *psb*A-*trn*H markers enhanced detection comprehensiveness through their distinct molecular characteristics (Lv et al., 2020; Zhu et al., 2022). ITS2’s broad taxonomic coverage enables wide species identification, while *psb*A-*trn*H’s specific amplification efficiency compensates for ITS2’s limitations in certain taxa. For instance, while ITS2 successfully identified most botanical materials, *psb*A-*trn*H specifically enabled the detection of *D. polystachya*, likely due to its conserved chloroplast genome regions that remain intact during processing.

The method’s enhanced sensitivity revealed both environmental contamination and authentication issues that conventional TLC (Raclariu et al., 2017b) and HPLC-MS (Raclariu et al., 2017a) techniques might overlook. Multiple high-abundance non-prescribed species from Fabaceae, Apiaceae, and Brassicaceae were frequently detected, raising significant quality control concerns. The primary sources may include field contamination during harvesting where non-target plants grow alongside medicinal plants, cross-contamination during processing on shared production lines, possible storage contamination as well as challenges in completely removing environmental DNA from raw materials. The presence of related species suggested potential cross-contamination or deliberate substitution during manufacturing (Liu et al., 2021b). Fungi were detected in the samples of some manufacturers (TY), suggesting improper preservation of botanical drugs in the production process. These contaminants could potentially affect therapeutic efficacy or safety through unexpected biological activities or allergenicity. These findings provide crucial insights into critical control points in the production chain that require strengthened monitoring (Liu et al., 2018). Notably, the identification of a nationally protected Class I species (*P. rockii*) in commercial samples demonstrated the technique’s value in conservation monitoring and regulatory compliance, highlighting the need for systematic oversight in raw material sourcing. Our findings thus emphasize the importance of implementing more rigorous quality control measures throughout the supply chain, including stricter source material authentication, improved cleaning procedures, dedicated production lines to prevent cross-contamination, and regular DNA metabarcoding screening as part of quality control protocols.

However, like any analytical method, DNA metabarcoding faces specific technical challenges, particularly regarding DNA integrity and processing effects. The failure to detect *P. cocos* and variable detection rates of *A. macrocephala* and *C. reticulata* revealed distinct DNA degradation patterns related to processing methods and taxonomic differences. *Poria cocos*, being a fungal ingredient, represents a taxonomic limitation of our plant-optimized markers. The ITS2 and psbA-trnH markers selected for this study are plant-specific, with the primers designed to preferentially amplify plant DNA. Fungal ingredients would require different marker regions and primers specifically optimized for fungal DNA, such as the full ITS region (ITS1-5.8S-ITS2) with fungal-specific primers. This highlights the need for multi-marker approaches when analyzing complex formulations containing ingredients from diverse taxonomic origins. For plant ingredients, processing-related DNA degradation presented variable challenges. The high-temperature processing of *A. macrocephala*, which typically involves stir-frying at 180–220°C, likely leads to DNA fragmentation, as thermal treatment is known to cause DNA degradation through denaturation (Karni et al., 2013). Additionally, oxidative metabolites in aged *C. reticulata* peel directly interfere with DNA stability (Li et al., 2024). These processing-specific DNA degradation mechanisms emphasize the importance of considering molecular integrity in quality control protocols (Li et al., 2023).

DNA metabarcoding should be integrated with other analytical approaches for comprehensive quality assessment. Its unique ability to detect both intended and unexpected ingredients makes it valuable for CCPP botanical drug authentication, despite limitations with processed materials. The integration with chemical analysis methods, particularly metabolomics, could provide both qualitative authentication and quantitative composition assessment, offering a more complete quality control solution (Coghlan et al., 2015; Gao et al., 2023). This integrated approach would provide complementary authentication perspectives, i.e., DNA metabarcoding identifies the biological origins, while metabolomics characterizes the bioactive metabolites. By detecting characteristic metabolites specific to each botanical ingredient, metabolomic profiling can confirm the presence of medicinally relevant compounds even when DNA is heavily degraded, enabling authentication at both the species level (DNA) and functional level (metabolites). Combined datasets could also help establish correlations between botanical ingredients and their metabolite profiles, potentially creating more robust authentication frameworks for complex formulations.

### 4.2 Technical optimization and methodological considerations for CCPP analysis

Previous research has demonstrated that modified extraction protocols can significantly improve DNA recovery from complex CCPP formulations (Arruda et al., 2017). Drawing on these findings, our protocol incorporated several refinements to address the complex nature of processed materials. The application of 75% ethanol pretreatment effectively reduced interference from polysaccharides and other processing-derived metabolites, while extended water bath incubation (1.5 h) enhanced DNA recovery from recalcitrant materials. These modifications were essential for improving DNA yield from highly processed botanical drugs, particularly those containing heavily processed or fungal materials. Though DNA recovery efficiency varies among ingredients due to their distinct processing methods and chemical compositions, the utilization of the AxyPrep DNA Gel Extraction Kit, combined with triplicate processing and pooling strategy, enabled consistent DNA isolation from complex CCPP matrices.

PCR bias emerged as a critical methodological challenge in our analysis, particularly evident in the dramatic fluctuations of *Z. jujuba* var. *spinosa* ASV counts. This phenomenon, well-documented in amplicon sequencing studies (Berry et al., 2011; Peng et al., 2015), manifested in our analysis as ASV count variations ranging from less than 10 to more than 90, suggesting substantial amplification preferences. These variations likely stem from differences in template GC content and secondary structure, factors known to influence PCR efficiency (Coissac et al., 2012). Recent advances in PCR optimization have suggested several promising approaches for bias mitigation. Modification of thermal cycling protocols and careful adjustment of reaction parameters have shown potential in reducing preferential amplification (Kanagawa, 2003). The development of standardized controls using mock communities has also demonstrated value in quantifying and correcting for amplification bias (McLaren et al., 2019).

The systematic validation of our methodology through standard controls and *P. quinquefolius*-spiked samples demonstrated robust qualitative detection capabilities, particularly for low-abundance ingredients. However, our findings revealed that quantitative applications require careful consideration of species-specific amplification efficiencies, highlighting the importance of appropriate controls and standardization procedures. The validation process provided crucial insights into both the strengths and limitations of our approach, establishing a foundation for method optimization.

Recent advancements have significantly expanded the analytical toolkit for CCPP authentication beyond conventional DNA barcoding. Although DNA barcoding has provided a foundational approach for botanical identification, its application to complex or processed formulations presents numerous challenges. Our metabarcoding approach demonstrated significantly greater sensitivity than conventional barcoding for detecting botanical ingredients in complex CCPP mixtures. However, alternative methodologies offer additional advantages for specific analytical challenges. For instance, shotgun metagenomics can eliminate amplification-related distortions while providing broader genomic coverage (Liu et al., 2021a), particularly beneficial for complex mixtures where PCR bias is problematic. For analyzing degraded DNA in processed materials, Single-Molecule Real-Time (SMRT) sequencing (Jia et al., 2017; Xin et al., 2018b) shows particular promise, though cost considerations currently limit its widespread adoption. TaqMan probe-based quantitative real-time PCR has emerged as another valuable technique for specific taxa like *Panax notoginseng* in complex CCPP formulations, offering greater sensitivity and quantitative capabilities (Lou et al., 2022). Unlike standard DNA barcoding, qPCR methods can detect target species at concentrations as low as 0.1%, making them particularly valuable for quality control in highly processed products where DNA is degraded. Complementary analytical approaches include multi-omics integration frameworks combining genomic, metabolomic, and chemical profiling data for holistic authentication (Wang et al., 2022). These integrative approaches have shown superior discriminatory power compared to single-method authentication, particularly for processed formulations where molecular integrity is compromised. Advanced computational methodologies have similarly transformed CCPP authentication. Machine learning algorithms, including deep learning and ensemble methods, have significantly improved pattern recognition capabilities for complex botanical mixtures (Chen and He, 2022; Magdas and Berghian-Grosan, 2023; Wang et al., 2024). These approaches can process multi-dimensional data from diverse analytical platforms, potentially overcoming the limitations of individual methods while providing more robust authentication frameworks. Recent innovations in molecular authentication include nucleotide signature-based identification strategies specifically optimized for processed materials (Zhang T. et al., 2022; Niu et al., 2024) and isothermal amplification methods for rapid authentication of complex materials (Sheu et al., 2023). While many of these technologies have primarily been validated in experimental settings, their translation to routine CCPP quality control represents a promising direction for future applications.

### 4.3 Future perspectives: Database development and bioinformatic integration

While DNA metabarcoding has become a powerful tool in environmental microbiome research with established specialized databases (SILVA, GreenGenes, and RDP) (McDonald et al., 2012; Quast et al., 2013; Cole et al., 2014), its application in TCM authentication faces significant database limitations. Currently, specialized platforms like the TCM DNA Barcode Identification System (Chen et al., 2014) and the DNA barcode databases from the Institute of Medicinal Plant Development (Chen et al., 2012) represent the primary resources for TCM identification. However, as demonstrated in our RSJPW analysis, these systems’ heavy reliance on public databases like NCBI limits their effectiveness for processed botanical materials. To enhance the practical application of DNA metabarcoding in CCPP quality control, several strategic advancements are crucial, such as development of comprehensive, validated reference databases specifically tailored for traditional medicinal plants and their common adulterants, iImproved bioinformatic pipelines optimized for highly processed materials with degraded DNA, standardized authentication protocols considering the unique challenges of complex formulations and integration of automated identification systems with regulatory databases. The processing of high-throughput sequencing data in CCPP analysis presents unique bioinformatic challenges beyond conventional environmental DNA studies. While QIIME 2 provides a robust analytical framework (Bolyen et al., 2019), our study revealed limitations in taxonomic assignment due to the scarcity of CCPP-specific reference databases. This issue is particularly evident in processed materials where DNA modifications can affect sequence matching accuracy. Recent studies have demonstrated improved authentication accuracy through specialized reference databases incorporating both raw and processed material sequences (Xin et al., 2018b).

The integration of bioinformatic tools with tiered analytical approaches represents a promising development direction in CCPP authentication. Recent work by Mück et al. (2024) demonstrated how combining DNA metabarcoding data with chemical profiles achieves improved authentication accuracy for complex formulations. This multi-analytical strategy provides both qualitative and quantitative insights, overcoming the limitations of conventional DNA barcoding which often proves insufficient for processed herbal materials. Furthermore, the development of automated analysis pipelines can streamline workflows and minimize reliance on high-performance computing resources (Fung et al., 2021; Dubois et al., 2022). These advances, coupled with expanding reference databases, will be crucial for improving the reliability and accessibility of DNA metabarcoding in CCPP authentication and could potentially create more robust authentication frameworks for complex formulations.

## 5 Conclusion

This study applied DNA metabarcoding to authenticate RSJPW, demonstrating both the capabilities and limitations of this approach in CCPP botnical drug authentication. Our dual-marker strategy successfully identified most prescribed ingredients while revealing authentication issues, including contamination and potential substitution in commercial products. However, processing-induced DNA degradation significantly affected detection rates for certain ingredients, particularly evident in *P. cocos* and heat-processed materials. These findings highlight the importance of considering processing effects in molecular authentication protocols.

While DNA metabarcoding offers advantages in multi-ingredient authentication, our results indicate that comprehensive CCPP quality assessment requires integration with complementary analytical methods. The systematic validation approach and optimized protocols developed in this study contribute to the methodological framework for complex botanical drug formulation analysis. Future improvements in CCPP-specific reference databases and bioinformatic tools, combined with chemical analysis methods (e.g., metabolomics), will be crucial for enhancing the practical application of DNA metabarcoding in CCPP botanical drug authentication.

## Data Availability

The raw sequencing data presented in this study are deposited in the NCBI Sequence Read Archive (SRA) repository under BioProject accession number PRJNA1242227.
