# Supplementary material for: DNA metabarcoding unveils authenticity and adulteration in commercial Chinese polyherbal preparations: Renshen Jianpi Wan as a critical case study
Source: Front Pharmacol. 2025 Apr 28;16:1584065. doi: 10.3389/fphar.2025.1584065 (PMC12066679; doi:10.3389/fphar.2025.1584065)
Supplement: Supplementary file 1 [file Table1.docx]

| Supplementary Table 1 Batch information and primer tags for reference and commercial samples | | | | | |
| --- | --- | --- | --- | --- | --- |
| Manufacturer Code | Batch Code | ITS2 | | *psb*A-*trn*H | |
|  |  | Forward | Reverse | Forward | Reverse |
| Reference samples | RF01 | ATCACG | TACAGC | ATCACG | TACAGC |
|  | RF02 | GAGATA | CTAGCT | GAGATA | CTAGCT |
|  | RF03 | CGCGGT | GAGTGG | CGCGGT | GAGTGG |
|  | RF04 | TCGGCA | AGTCAA | TCGGCA | AGTCAA |
|  | RF05 | AGTTCC | TGAATG | AGTTCC | TGAATG |
|  | RF06 | GTACTT | CCAGCT | GTACTT | CCAGCT |
|  | RF07 | CAGATC | GTGAAA | CAGATC | GTGAAA |
|  | RF08 | TAATCG | ACTTGA | TAATCG | ACTTGA |
| FC | FC01 | AGTTCC | TGAATG | AGTTCC | TGAATG |
|  | FC02 | GTACTT | CCAGCT | GTACTT | CCAGCT |
|  | FC03 | CAGATC | GTGAAA | CAGATC | GTGAAA |
|  | FC04 | TAATCG | ACTTGA | TAATCG | ACTTGA |
|  | FC05 | ATCACG | TACAGC | ATCACG | TACAGC |
| DR | DR01 | GAGATA | CTAGCT | GAGATA | CTAGCT |
|  | DR02 | CGCGGT | GAGTGG | CGCGGT | GAGTGG |
|  | DR03 | TCGGCA | AGTCAA | TCGGCA | AGTCAA |
|  | DR04 | ATCGTA | TCTACC | ATCGTA | TCTACC |
|  | DR05 | GGTTGT | CGATGT | GGTTGT | CGATGT |
| KM | KM01 | CCACAA | GCCAAT | CCACAA | GCCAAT |
|  | KM02 | TCGAAG | AGTGGA | TCGAAG | AGTGGA |
|  | KM03 | ACAGTG | TTCAGA | ACAGTG | TTCAGA |
|  | KM04 | GTTGAA | CATCGT | GTTGAA | CATCGT |
|  | KM05 | CGTACG | GCCGCG | CGTACG | GCCGCG |
|  | KM06 | TCCCGA | ATGTCA | TCCCGA | ATGTCA |
| YH | YH01 | ACCTAA | TCATTC | ACCTAA | TCATTC |
|  | YH02 | GTTTCG | CTATAC | GTTTCG | CTATAC |
|  | YH03 | CGGAAT | GGTAGC | CGGAAT | GGTAGC |
|  | YH04 | TAACGA | ATATGT | TAACGA | ATATGT |
|  | YH05 | AGAGTA | TTAGGC | AGAGTA | TTAGGC |
|  | YH06 | GGAAGA | CTCAGA | GGAAGA | CTCAGA |
| ML | ML01 | CTTCCA | GCCTTA | CTTCCA | GCCTTA |
|  | ML02 | TGACCA | ATGCCT | TGACCA | ATGCCT |
|  | ML03 | AGTTCC | TGAATG | AGTTCC | TGAATG |
|  | ML04 | GTACTT | CCAGCT | GTACTT | CCAGCT |
|  | ML05 | CAGATC | GTGAAA | CAGATC | GTGAAA |
| LX | LX01 | TAATCG | ACTTGA | TAATCG | ACTTGA |
|  | LX02 | ATCACG | TACAGC | ATCACG | TACAGC |
|  | LX03 | GAGATA | CTAGCT | GAGATA | CTAGCT |
|  | LX04 | CGCGGT | GAGTGG | CGCGGT | GAGTGG |
|  | LX05 | TCGGCA | AGTCAA | TCGGCA | AGTCAA |
|  | LX06 | ATCGTA | TCTACC | ATCGTA | TCTACC |
| TY | TY01 | GGTTGT | CGATGT | GGTTGT | CGATGT |
|  | TY02 | CCACAA | GCCAAT | AGTTCC | TGAATG |
|  | TY03 | TCGAAG | AGTGGA | GTACTT | CCAGCT |
|  | TY04 | ACAGTG | TTCAGA | CAGATC | GTGAAA |
|  | TY05 | GTTGAA | CATCGT | TAATCG | ACTTGA |
| PJ | PJ01 | CGTACG | GCCGCG | CGTACG | GCCGCG |
|  | PJ02 | TCCCGA | ATGTCA | TCCCGA | ATGTCA |
|  | PJ03 | ACCTAA | TCATTC | ACCTAA | TCATTC |
|  | PJ04 | GTTTCG | CTATAC | GTTTCG | CTATAC |
|  | PJ05 | CGGAAT | GGTAGC | CGGAAT | GGTAGC |
| ZJ | ZJ01 | TAACGA | ATATGT | TAACGA | ATATGT |
|  | ZJ02 | AGAGTA | TTAGGC | AGAGTA | TTAGGC |
|  | ZJ03 | GGAAGA | CTCAGA | GGAAGA | CTCAGA |
| ZD | ZD01 | CTTCCA | GCCTTA | CTTCCA | GCCTTA |
|  | ZD02 | TGACCA | ATGCCT | TGACCA | ATGCCT |
| YS | YS01 | AGTTCC | TGAATG | AGTTCC | TGAATG |
|  | YS02 | GTACTT | CCAGCT | GTACTT | CCAGCT |
| TR | TR01 | AGTTCC | TGAATG | AGTTCC | TGAATG |
|  | TR02 | GTACTT | CCAGCT | GTACTT | CCAGCT |
|  | TR03 | CAGATC | GTGAAA | CAGATC | GTGAAA |
|  | TR04 | TAATCG | ACTTGA | TAATCG | ACTTGA |
|  | TR05 | ATCACG | TACAGC | ATCACG | TACAGC |
|  | TR06 | GAGATA | CTAGCT | GAGATA | CTAGCT |
