# Supplementary material for: DNA metabarcoding unveils authenticity and adulteration in commercial Chinese polyherbal preparations: Renshen Jianpi Wan as a critical case study
Source: Front Pharmacol. 2025 Apr 28;16:1584065. doi: 10.3389/fphar.2025.1584065 (PMC12066679; doi:10.3389/fphar.2025.1584065)
Supplement: Supplementary file 2 [file Table8.docx]

| Supplementary Table 8 Non-prescribed species detected in commercial RSJPW samples based on ITS2 sequences | | | | | | |
| --- | --- | --- | --- | --- | --- | --- |
| Family | Latin  name | Reads  No. | ASV  No. | Sample  No. | Detection  frequency (%) | Possible source  category |
| [Altingiaceae](https://www.iplant.cn/info/Altingiaceae) | *Liquidambar formosana* Hance | 27 | 1 | 1 | 1.79 | Wild plant |
| Amaranthaceae | *Chenopodiastrum murale* (L.) S.Fuentes, Uotila & Borsch | 34 | 1 | 2 | 3.57 | Wild plant |
|  | *Hymenocallis littoralis* (Jacq.) Salisb. | 987 | 20 | 6 | 10.71 | Wild plant |
|  | *Allium sativum* L. | 902 | 5 | 9 | 16.07 | Food crop |
|  | *Allium altaicum* Pall. | 714 | 1 | 3 | 5.36 | Food crop |
|  | *Allium fistulosum* L. | 73 | 2 | 2 | 3.57 | Food crop |
|  | *Allium cepa* L. | 192 | 6 | 6 | 10.71 | Food crop |
| Anacardiaceae | *Mangifera indica* L. | 192 | 3 | 1 | 1.79 | Food crop |
| Apiaceae | *Peucedanum caespitosum* H. Wolff | 9,926 | 17 | 6 | 10.71 | Medicinal plant |
|  | *Peucedanum praeruptorum* Dunn | 4,776 | 11 | 8 | 14.29 | Medicinal plant |
|  | ***Angelica acutiloba* (Siebold & Zucc.) Kitag.** | 12 | 1 | 1 | 1.79 | Medicinal plant |
|  | *Bupleurum chinense* Franch. | 13 | 1 | 2 | 3.57 | Medicinal plant |
|  | *Foeniculum vulgare* Mill*.* | 164 | 3 | 3 | 5.36 | Food crop |
|  | *Ligusticum acuminatum* Franch. | 17 | 1 | 1 | 1.79 | Medicinal plant |
|  | *Peucedanum officinale* L. | 47 | 1 | 1 | 1.79 | Medicinal plant |
|  | *Cnidium monnieri* (L.) Spreng. | 193 | 5 | 4 | 7.14 | Medicinal plant |
|  | *Hymenidium giraldii* (Diels) Pimenov & Kljuykov | 19 | 1 | 4 | 7.14 | Medicinal plant |
|  | *Peucedanum wawrae* (H. Wolff) Su | 186 | 4 | 3 | 5.36 | Medicinal plant |
|  | *Cuminum cyminum* L. | 16 | 1 | 1 | 1.79 | Food crop |
| Araliaceae | ***Panax quinquefolius* L*.*** | 95 | 1 | 2 | 3.57 | Medicinal plant |
|  | ***Panax japonicus* (T. Nees) C. A. Meyer** | 11 | 1 | 1 | 1.79 | Medicinal plant |
| Asteraceae | *Artemisia argyi* H. Lév. & Vaniot | 4,707 | 10 | 1 | 1.79 | Medicinal plant |
|  | *Lactuca sativa* L*.* | 68 | 1 | 4 | 7.14 | Food crop |
| Berberidaceae | *Epimedium borealiguizhouense* S. Z. He & Y. K. Yang | 119 | 2 | 3 | 5.36 | Medicinal plant |
| Betulaceae | *Alnus nepalensis* D. Don | 4,351 | 10 | 25 | 44.64 | Wild plant |
|  | *Ostryopsis nobilis* Balf. f. & W. W. Sm. | 25 | 1 | 1 | 1.79 | Wild plant |
|  | *Alnus trabeculosa* Hand.-Mazz. | 66 | 1 | 1 | 1.79 | Wild plant |
|  | *Betula alnoides* Buch.-Ham. ex D. Don | 30 | 1 | 3 | 5.36 | Wild plant |
| Brassicaceae | *Brassica napus* L*.* | 18,182 | 58 | 15 | 26.79 | Food crop |
|  | *Raphanus sativus* L*.* | 5,067 | 9 | 14 | 25.00 | Food crop |
|  | *Descurainia sophia* (L.) Webb ex Prantl | 33 | 2 | 1 | 1.79 | Food crop |
|  | *Mutarda nigra* (L.) Bernh. | 40 | 2 | 4 | 7.14 | Medicinal plant |
|  | *Brassica juncea* (L.) Czern. | 156 | 7 | 1 | 1.79 | Food crop |
|  | *Brassica carinata* A.Braun | 20 | 1 | 2 | 3.57 | Wild plant |
| Campanulaceae | ***Codonopsis pilosula* (Franch.) Nannf.** | 116 | 1 | 2 | 3.57 | Medicinal plant |
| [Cannabaceae](https://www.iplant.cn/info/Cannabaceae) | *Celtis koraiensis* Nakai | 31 | 1 | 2 | 3.57 | Wild plant |
|  | *Humulus scandens* (Lour.) Merr. | 46 | 1 | 2 | 3.57 | Wild plant |
| Caprifoliaceae | *Dipsacus asper* Wall*.* | 33 | 1 | 1 | 1.79 | Medicinal plant |
| [Celastraceae](https://www.iplant.cn/info/Celastraceae) | *Celastrus gemmatus* Loes. | 15 | 1 | 1 | 1.79 | Wild plant |
| Convolvulaceae | *Cuscuta australis* R. Br. | 3,977 | 7 | 17 | 30.36 | Medicinal plant |
|  | *Ipomoea hederacea* Jacq. | 408 | 7 | 10 | 17.86 | Medicinal plant |
|  | *Ipomoea nil* (L.) Roth | 10 | 1 | 1 | 1.79 | Medicinal plant |
|  | *Ipomoea purpurea* (L.) Roth | 18 | 1 | 1 | 1.79 | Medicinal plant |
| Cucurbitaceae | *Cucurbita moschata* (Duch. ex Lam.) Duch. ex Poir. | 11,870 | 15 | 22 | 39.29 | Food crop |
|  | *Cucumis sativus* L. | 3,122 | 12 | 3 | 5.36 | Food crop |
|  | *Luffa acutangula* (L.) Roxb. | 402 | 5 | 6 | 10.71 | Food crop |
|  | *Cucumis melo* L. | 84 | 2 | 6 | 10.71 | Food crop |
|  | *Citrullus lanatus* (Thunb.) Matsum. & Nakai | 46 | 1 | 2 | 3.57 | Food crop |
| Euphorbiaceae | *Euphorbia pulcherrima* Willd. ex Klotzsch | 11 | 1 | 1 | 1.79 | Wild plant |
|  | *Macaranga denticulata* (Blume) Müll. Arg. | 187 | 5 | 7 | 12.50 | Wild plant |
| Fabaceae | *Arachis villosa* Benth. | 755 | 6 | 5 | 8.93 | Food crop |
|  | ***Hedysarum polybotrys* Hand.-Mazz.** | 343 | 3 | 5 | 8.93 | Medicinal plant |
|  | *Lablab purpureus* (L.) Sweet | 116 | 4 | 4 | 7.14 | Food crop |
|  | *Phaseolus vulgaris* L. | 26 | 2 | 2 | 3.57 | Food crop |
|  | *Vicia faba* L. | 111 | 1 | 2 | 3.57 | Food crop |
|  | *Senna obtusifolia* (L.) H. S. Irwin & Barneby | 18 | 1 | 2 | 3.57 | Medicinal plant |
|  | *Glycine max* (L.) Merr. | 21 | 1 | 5 | 8.93 | Food crop |
|  | *Glycyrrhiza uralensis* Fisch. | 137 | 2 | 6 | 10.71 | Medicinal plant |
|  | *Styphnolobium japonicum* (L.) Schott | 113 | 2 | 5 | 8.93 | Medicinal plant |
|  | *Vigna unguiculata* (L.) Walp. | 89 | 2 | 5 | 8.93 | Food crop |
|  | *Spatholobus suberectus* Dunn | 152 | 3 | 2 | 3.57 | Medicinal plant |
|  | *Gueldenstaedtia stenophylla* Bunge | 21 | 1 | 1 | 1.79 | Medicinal plant |
|  | *Bauhinia purpurea* L. | 29 | 1 | 1 | 1.79 | Wild plant |
|  | *Robinia pseudoacacia* L. | 224 | 3 | 6 | 10.71 | Wild plant |
|  | *Quercus suber* L. | 195 | 3 | 3 | 5.36 | Wild plant |
|  | *Quercus cerris* L. | 15 | 1 | 1 | 1.79 | Wild plant |
| Juglandaceae | *Engelhardia spicata* Lesch. ex Blume | 59 | 1 | 1 | 1.79 | Medicinal plant |
| Lamiaceae | *Nepeta cataria* L. | 85 | 2 | 6 | 10.71 | Medicinal plant |
| Loranthaceae | *Scurrula parasitica* L. | 47 | 2 | 3 | 5.36 | Medicinal plant |
| Lythraceae | *Punica granatum* L. | 150 | 2 | 3 | 5.36 | Food crop |
|  | *Cuphea hyssopifolia* Kunth | 163 | 2 | 4 | 7.14 | Wild plant |
| [Malvaceae](https://www.iplant.cn/info/Malvaceae) | *Urena lobata* L. | 127 | 4 | 4 | 7.14 | Wild plant |
|  | *Gossypium hirsutum* L. | 34 | 1 | 1 | 1.79 | Wild plant |
| [Meliaceae](https://www.iplant.cn/info/Meliaceae) | *Melia azedarach* L. | 38 | 2 | 5 | 8.93 | Wild plant |
| Moraceae | *Morus alba* L. | 24 | 1 | 3 | 5.36 | Medicinal plant |
| [Nelumbonaceae](https://www.iplant.cn/info/Nelumbonaceae) | *Nelumbo nucifera* Gaertn*.* | 95 | 2 | 1 | 1.79 | Food crop |
| [Oleaceae](https://www.iplant.cn/info/Oleaceae) | *Forsythia suspensa* (Thunb.) Vahl | 34 | 1 | 2 | 3.57 | Medicinal plant |
| Paeoniaceae | *Paeonia* × *suffruticosa* Andrews | 1,040 | 3 | 3 | 5.36 | Medicinal plant |
|  | *Paeonia lactiflora* Pall. | 726 | 3 | 14 | 25.00 | Medicinal plant |
| Pinaceae | *Pinus tabuliformis* Carrière | 1,438 | 6 | 12 | 21.43 | Wild plant |
|  | *Pinus strobus* L. | 213 | 8 | 5 | 8.93 | Wild plant |
|  | *Pinus massoniana* Lamb. | 234 | 9 | 3 | 5.36 | Wild plant |
|  | *Tsuga chinensis* (Franch.) E. Pritz. | 10 | 1 | 1 | 1.79 | Wild plant |
|  | *Pinus sibirica* (Ledeb.) Turcz. | 156 | 4 | 2 | 3.57 | Wild plant |
|  | *Tsuga dumosa* (D. Don) Eichler | 28 | 1 | 1 | 1.79 | Wild plant |
| [Pittosporaceae](https://www.iplant.cn/info/Pittosporaceae) | *Pittosporum brevicalyx* (Oliv.) Gagnep. | 59 | 1 | 1 | 1.79 | Wild plant |
| [Plantaginaceae](https://www.iplant.cn/info/Plantaginaceae) | *Plantago major* L. | 85 | 1 | 3 | 5.36 | Medicinal plant |
| Poaceae | *Setaria viridis* (L.) P. Beauv. | 4,356 | 2 | 6 | 10.71 | Wild plant |
|  | *Triticum aestivum* L. | 3,938 | 9 | 24 | 42.86 | Food crop |
|  | *Festuca pratensis* Huds. | 13 | 1 | 1 | 1.79 | Wild plant |
|  | *Sorghum halepense* (L.) Pers. | 149 | 3 | 7 | 12.50 | Wild plant |
|  | *Poa annua* L. | 28 | 1 | 2 | 3.57 | Wild plant |
| Podocarpaceae | *Podocarpus macrophyllus* (Thunb.) Sweet | 26 | 1 | 1 | 1.79 | Wild plant |
| Ranunculaceae | *Clematis tangutica* (Maxim.) Korsh. | 70 | 1 | 1 | 1.79 | Medicinal plant |
| Rhamnaceae | ***Ziziphus mauritiana* Lam.** | 551 | 8 | 2 | 3.57 | Medicinal plant |
| [Rosaceae](https://www.iplant.cn/info/Rosaceae) | *Argentina anserina* (L.) Rydb. | 26 | 2 | 2 | 3.57 | Medicinal plant |
|  | *Prunus avium* (L.) L. | 276 | 2 | 2 | 3.57 | Food crop |
|  | *Prunus geniculata* R.M.Harper | 19 | 1 | 1 | 1.79 | Wild plant |
|  | *Prunus persica* (L.) Batsch | 101 | 3 | 3 | 5.36 | Food crop |
|  | *Prunus sibirica* L. | 30 | 1 | 1 | 1.79 | Medicinal plant |
|  | *Potentilla cuneata* Wall. ex Lehm. | 15 | 1 | 1 | 1.79 | Wild plant |
|  | *Prunus armeniaca* L. | 64 | 4 | 5 | 8.93 | Food crop |
|  | ***Citrus sinensis* (L.) Osbeck** | 16 | 1 | 2 | 3.57 | Food crop |
|  | *Tetradium ruticarpum* (A. Juss.) T. G. Hartley | 16 | 1 | 1 | 1.79 | Medicinal plant |
|  | *Zanthoxylum armatum* DC. | 108 | 1 | 1 | 1.79 | Medicinal plant |
| Salicaceae | *Salix matsudana* Koidz. | 509 | 2 | 8 | 14.29 | Wild plant |
|  | *Populus nigra* L. | 305 | 6 | 4 | 7.14 | Wild plant |
|  | *Populus deltoides* W.Bartram ex Marshall | 118 | 3 | 5 | 8.93 | Wild plant |
|  | *Salix pentandra* L. | 261 | 2 | 5 | 8.93 | Wild plant |
|  | *Salix vestita* Pursh | 16 | 1 | 1 | 1.79 | Wild plant |
| Sapindaceae | *Dimocarpus longan* Lour. | 62 | 1 | 1 | 1.79 | Food crop |
| Solanaceae | *Solanum lycopersicum* L. | 33 | 1 | 1 | 1.79 | Food crop |
|  | *Solanum nigrum* L. | 84 | 1 | 3 | 5.36 | Medicinal plant |
|  | *Nicotiana tabacum* L. | 136 | 2 | 1 | 1.79 | Medicinal plant |
| Ulmaceae | *Ulmus pumila* L. | 19 | 1 | 3 | 5.36 | Wild plant |
| Verbenaceae | *Verbena officinalis* L. | 23,911 | 36 | 14 | 25.00 | Medicinal plant |
|  | *Verbena bracteata* Lag. & Rodr. | 15 | 1 | 1 | 1.79 | Medicinal plant |
| [Zingiberaceae](https://www.iplant.cn/info/Zingiberaceae) | ***Amomum compactum* Soland ex Maton** | 65 | 1 | 1 | 1.79 | Medicinal plant |
| Bacillaceae | *Bacillus altitudinis* | 275 | 3 | 4 | 7.14 | / |
| Dimargaritaceae | *Dimargaris bacillispora* | 21 | 1 | 1 | 1.79 | / |

Note: Detection frequency = (Number of samples where the species is detected / Total number of samples) × 100%; species in bold represent adulterants or substitutes of the prescribed ingredients as documented in the literature.
