# Supplementary material for: DNA metabarcoding unveils authenticity and adulteration in commercial Chinese polyherbal preparations: Renshen Jianpi Wan as a critical case study
Source: Front Pharmacol. 2025 Apr 28;16:1584065. doi: 10.3389/fphar.2025.1584065 (PMC12066679; doi:10.3389/fphar.2025.1584065)
Supplement: Supplementary file 3 [file Table7.docx]

| Supplementary Table 7 Number of ASV reads for detected species in commercial RSJPW samples based on *psb*A-*trn*H sequences | | | | | | |
| --- | --- | --- | --- | --- | --- | --- |
| Ingredient  Batch code | Ginseng Radix et Rhizoma | Dioscoreae  Rhizoma | Astragali  Radix | Angelicae Sinensis  Radix | Ziziphi Spinosae  Semen | Polygalae Radix |
| TR01 | -- | 10 | -- | 554 | 26,615 | -- |
| TR02 | 59 | -- | -- | 142 | 23,401 | -- |
| TR03 | 13 | -- | -- | 137 | 22,786 | -- |
| TR04 | 25 | 11 | 2 | 397 | 24,851 | -- |
| TR05 | 15 | -- | -- | 3,069 | 23,880 | -- |
| TR06 | 47 | -- | 5 | 1,785 | 23,263 | -- |
| FC01 | -- | -- | -- | 383 | 24,903 | -- |
| FC02 | -- | 20 | -- | 93 | 22,412 | -- |
| FC03 | 2 | 17 | -- | 37 | 23,288 | -- |
| FC04 | -- | -- | -- | 32 | 22,166 | -- |
| FC05 | -- | 45 | -- | 92 | 21,947 | -- |
| DR01 | 22 | 8 | -- | 164 | 22,694 | -- |
| DR02 | 12 | 959 | 3,058 | 15,348 | 6,313 | -- |
| DR03 | 15 | -- | -- | 478 | 22,814 | -- |
| DR04 | -- | 3 | 4 | 2,970 | 18,667 | -- |
| DR05 | 69 | 92 | 8 | 8,152 | 19,124 | -- |
| KM01 | 4 | 171 | 33 | 449 | 23,911 | -- |
| KM02 | -- | 209 | 23 | 315 | 20,827 | -- |
| KM03 | -- | 262 | 35 | 233 | 27,252 | -- |
| KM04 | 2 | 141 | 81 | 588 | 21,134 | -- |
| KM05 | 2 | 1,431 | 85 | 1,432 | 22,401 | -- |
| KM06 | -- | 184 | 12 | 683 | 24,931 | -- |
| YH01 | 145 | 6,060 | 2,681 | 13,777 | 979 | -- |
| YH02 | 39 | 7,006 | 3,232 | 10,844 | 1,110 | -- |
| YH03 | 41 | 10,557 | 6,886 | 9,111 | 3,056 | 4 |
| YH04 | 124 | 510 | 3,434 | 9,655 | 8,885 | -- |
| YH05 | 34 | 303 | 249 | 5,940 | 19,486 | -- |
| YH06 | 15 | 5,559 | 6,631 | 10,035 | 6,863 | -- |
| ML01 | 77 | 283 | 46 | 3,338 | 20,452 | -- |
| ML02 | 44 | 441 | 165 | 6,483 | 16,964 | 12 |
| ML03 | 45 | 1,197 | 75 | 5,909 | 18,857 | -- |
| ML04 | 37 | 1,105 | 39 | 4,444 | 16,064 | -- |
| ML05 | 64 | 2,103 | 92 | 7,459 | 15,715 | -- |
| LX01 | 315 | 2,091 | 196 | 20,931 | 12,200 | -- |
| LX02 | 200 | 1,807 | 178 | 14,696 | 12,734 | -- |
| LX03 | 85 | 127 | 126 | 7,324 | 16,865 | -- |
| LX04 | 52 | 99 | 235 | 4,145 | 26,271 | -- |
| LX05 | 27 | 586 | 52 | 10,369 | 15,002 | -- |
| LX06 | 58 | 372 | 80 | 7,845 | 16,545 | -- |
| TY01 | -- | -- | 98 | 4,031 | 12,139 | -- |
| TY02 | -- | -- | -- | 10,752 | -- | -- |
| TY03 | -- | -- | -- | 14,310 | -- | -- |
| TY04 | -- | -- | -- | 21,284 | -- | -- |
| TY05 | -- | -- | -- | 21,550 | -- | -- |
| PJ01 | 17 | 128 | -- | 3,002 | 24,352 | -- |
| PJ02 | 25 | 224 | -- | 3,030 | 19,928 | -- |
| PJ03 | 4 | 131 | -- | 2,025 | 19,374 | -- |
| PJ04 | 4 | 149 | 7 | 3,475 | 18,993 | -- |
| PJ05 | 40 | 141 | 7 | 4,361 | 23,039 | -- |
| ZJ01 | 17 | 85 | 21 | 6,101 | 16,717 | -- |
| ZJ02 | 78 | 24 | 209 | 5,042 | 22,463 | -- |
| ZJ03 | 180 | 69 | 65 | 4,624 | 23,219 | -- |
| ZD01 | 222 | 67 | 91 | 2,431 | 25,147 | -- |
| ZD02 | 54 | 371 | 40 | 2,154 | 20,887 | -- |
| YS01 | 11 | 6,513 | 173 | 3,798 | 25,519 | -- |
| YS02 | 15 | 15,554 | 1,117 | 7,252 | 1,229 | -- |
| Total | 2,356 | 67,225 | 29,571 | 309,060 | 970,634 | 16 |
| Relative  abundance (%) | 0.16 | 4.54 | 2.00 | 20.87 | 65.54 | 0.0011 |

Note: --: No reads was detected for this species in this sample; Relative abundance = (Number of ASV reads for the species / Total number of reads) × 100%
