# Supplementary material for: DNA metabarcoding unveils authenticity and adulteration in commercial Chinese polyherbal preparations: Renshen Jianpi Wan as a critical case study
Source: Front Pharmacol. 2025 Apr 28;16:1584065. doi: 10.3389/fphar.2025.1584065 (PMC12066679; doi:10.3389/fphar.2025.1584065)
Supplement: Supplementary file 4 [file Table9.docx]

| Supplementary Table 9 Non-prescribed species detected in commercial RSJPW samples based on *psb*A-*trn*H sequences | | | | | | |
| --- | --- | --- | --- | --- | --- | --- |
| Family | Latin  name | Reads  No. | ASV  No. | Sample  No. | Detection  frequency (%) | Possible source  category |
| Amaranthaceae | *Amaranthus tricolor* L*.* | 107 | 1 | 4 | 7.14 | Food crop |
| Anacardiaceae | *Mangifera indica* L. | 917 | 1 | 3 | 5.36 | Food crop |
| Apiaceae | *Peucedanum praeruptorum* Dunn | 333 | 2 | 2 | 3.57 | Medicinal plant |
|  | *Angelica polymorpha* Maxim. | 71 | 4 | 7 | 12.50 | Medicinal plant |
|  | *Peucedanum harry-smithii* Fedde ex H. Wolff | 80 | 2 | 2 | 3.57 | Medicinal plant |
|  | *Foeniculum vulgare* Mill. | 131 | 1 | 1 | 1.79 | Food crop |
| Asteraceae | *Echinops latifolius* Tausch | 318 | 2 | 5 | 8.93 | Medicinal plant |
|  | *Chrysanthemum morifolium* Ramat. | 245 | 1 | 1 | 1.79 | Medicinal plant |
| Berberidaceae | *Epimedium chlorandrum* Stearn | 37 | 1 | 1 | 1.79 | Medicinal plant |
| Brassicaceae | *Brassica juncea* (L.) Czern. | 34 | 1 | 1 | 1.79 | Food crop |
|  | *Raphanus sativus* L. | 281 | 4 | 8 | 14.29 | Food crop |
| Campanulaceae | ***Codonopsis pilosula* (Franch.) Nannf.** | 402 | 3 | 6 | 10.71 | Medicinal plant |
| Caryophyllaceae | *Stellaria vestita* Kurz | 49 | 1 | 6 | 10.71 | Wild plant |
| Elaeagnaceae | *Hippophae rhamnoides* L. | 22 | 1 | 2 | 3.57 | Food crop |
| Ericaceae | *Pyrola calliantha* Andres | 84 | 1 | 1 | 1.79 | Medicinal plant |
| Fabaceae | *Arachis hypogaea* L. | 62,146 | 9 | 36 | 64.29 | Food crop |
|  | *Acacia mearnsii* De Wild. | 387 | 1 | 1 | 1.79 | Wild plant |
|  | *Pisum sativum* L. | 183 | 1 | 4 | 7.14 | Food crop |
|  | ***Hedysarum polybotrys* Hand.-Mazz*.*** | 66 | 2 | 2 | 3.57 | Medicinal plant |
|  | *Melilotus albus* Desr. | 19 | 1 | 1 | 1.79 | Wild plant |
|  | *Phaseolus vulgaris* L*.* | 19 | 1 | 2 | 3.57 | Food crop |
|  | *Phaseolus coccineus* L. | 20 | 1 | 1 | 1.79 | Food crop |
|  | *Styphnolobium japonicum* (L.) Schott | 42 | 1 | 2 | 3.57 | Medicinal plant |
|  | *Vigna unguiculata* (L.) Walp. | 43 | 2 | 4 | 7.14 | Food crop |
|  | *Wisteria villosa* Rehder | 26 | 1 | 1 | 1.79 | Wild plant |
|  | *Glycyrrhiza glabra* L. | 15 | 1 | 3 | 5.36 | Medicinal plant |
|  | *Glycine soja* Siebold & Zucc*.* | 24 | 1 | 2 | 3.57 | Wild plant |
|  | *Glycine max* (L.) Merr. | 31 | 1 | 6 | 10.71 | Food crop |
| Juglandaceae | *Juglans mandshurica* Maxim*.* | 33 | 1 | 1 | 1.79 | Wild plant |
| Lamiaceae | *Lamium amplexicaule* L. | 75 | 1 | 1 | 1.79 | Medicinal plant |
|  | *Scutellaria baicalensis* Georgi | 30 | 1 | 1 | 1.79 | Medicinal plant |
| Lathyrus | *Lathyrus quinquenervius* (Miq.) Litv. | 24 | 1 | 1 | 1.79 | Food crop |
| Paeoniaceae | *Paeonia rockii* (S. G. Haw & Lauener) T. Hong & J. J. Li | 3,135 | 3 | 6 | 10.71 | Medicinal plant |
|  | *Paeonia jishanensis* T. Hong & W. Z. Zhao | 670 | 1 | 2 | 3.57 | Medicinal plant |
|  | *Paeonia lactiflora* Pall. | 458 | 1 | 8 | 14.29 | Medicinal plant |
| Plantaginaceae | *Veronica persica* Poir. | 12 | 1 | 2 | 3.57 | Wild plant |
|  | *Plantago depressa* Willd. | 104 | 3 | 2 | 3.57 | Medicinal plant |
| Polygonaceae | *Polygonum multiflorum* Thunb. | 14,051 | 13 | 9 | 16.07 | Medicinal plant |
|  | *Persicaria neofiliformis* (Nakai) Ohki | 30 | 1 | 1 | 1.79 | Medicinal plant |
|  | *Rumex nepalensis* Spreng*.* | 153 | 4 | 5 | 8.93 | Wild plant |
|  | *Knorringia sibirica* (Laxm.) Tzvelev | 59 | 1 | 4 | 7.14 | Wild plant |
| Portulacaceae | *Portulaca oleracea* L. | 14 | 1 | 1 | 1.79 | Medicinal plant |
| Ranunculaceae | *Aconitum vilmorinianum* Kom. | 13 | 1 | 1 | 1.79 | Medicinal plant |
| Rhamnaceae | ***Ziziphus mauritiana* Lam.** | 205 | 5 | 9 | 16.07 | Medicinal plant |
| Rosaceae | *Prunus cerasoides* Buch.-Ham. ex D. Don | 14 | 1 | 2 | 3.57 | Wild plant |
|  | *Prunus salicina* Lindl. | 17 | 1 | 2 | 3.57 | Food crop |
| Rubiaceae | *Gardenia jasminoides* J. Ellis | 17 | 1 | 1 | 1.79 | Medicinal plant |
| Salicaceae | *Salix alba* L*.* | 9,593 | 3 | 27 | 48.21 | Wild plant |
|  | *Populus deltoides* W.Bartram ex Marshall | 2,087 | 1 | 4 | 7.14 | Wild plant |
|  | *Salix pentandra* L*.* | 355 | 4 | 7 | 12.50 | Wild plant |
|  | *Populus szechuanica* C. K. Schneid*.* | 16 | 1 | 1 | 1.79 | Wild plant |
|  | *Salix babylonica* L*.* | 56 | 1 | 1 | 1.79 | Wild plant |
| Schisandraceae | *Schisandra chinensis* (Turcz.) Baill. | 18 | 1 | 1 | 1.79 | Medicinal plant |
| Ulmaceae | *Ulmus minor* Mill. | 168 | 1 | 7 | 12.50 | Wild plant |
| Verbenaceae | *Verbena officinalis* L. | 4,562 | 1 | 7 | 12.50 | Medicinal plant |

Note: Detection frequency = (Number of samples where the species is detected / Total number of samples) × 100%; species in bold represent adulterants or substitutes of the prescribed ingredients as documented in the literature.
