# Supplementary material for: DNA metabarcoding unveils authenticity and adulteration in commercial Chinese polyherbal preparations: Renshen Jianpi Wan as a critical case study
Source: Front Pharmacol. 2025 Apr 28;16:1584065. doi: 10.3389/fphar.2025.1584065 (PMC12066679; doi:10.3389/fphar.2025.1584065)
Supplement: Supplementary file 5 [file Table2.docx]

| Supplementary Table 2 Primer sequences and PCR reaction conditions | | | |
| --- | --- | --- | --- |
| Amplified  Region | Primer | Sequence (5'-3') | Condition |
| ITS2 | ITS2F | ATGCGATACTTGGTGTGAAT | 95℃, 3 min; 40 cycles: 95℃, 30 s; 60℃, 30 s; 72℃, 45 s; 72℃, 10 min. |
|  | ITS3R | GACGCTTCTCCAGACTACAAT |  |
| *psb*A-*trn*H | *psb*AF | GTTATGCATGAACGTAATGCTC | 95℃, 3 min; 35 cycles: 95℃, 30 s; 55℃, 30 s; 72℃, 45 s; 72℃, 10 min. |
|  | *trn*HR | CGCGCATGGTGGATTCACAATCC |  |
