# Supplementary material for: DNA metabarcoding unveils authenticity and adulteration in commercial Chinese polyherbal preparations: Renshen Jianpi Wan as a critical case study
Source: Front Pharmacol. 2025 Apr 28;16:1584065. doi: 10.3389/fphar.2025.1584065 (PMC12066679; doi:10.3389/fphar.2025.1584065)
Supplement: Supplementary file 6 [file Table5.docx]

| Supplementary Table 5 Number of ASVs for detected species in commercial RSJPW samples based on *psb*A-*trn*H sequences | | | | | | | | |
| --- | --- | --- | --- | --- | --- | --- | --- | --- |
| Ingredient  Batch code | Ginseng Radix et Rhizoma | Dioscoreae  Rhizoma | Astragali  Radix | Angelicae Sinensis  Radix | Ziziphi Spinosae  Semen | Polygalae Radix | Other species | Total |
| TR01 | -- | 1 | -- | 1 | 15 | -- | 1 | 18 |
| TR02 | 1 | -- | -- | 1 | 14 | -- | 3 | 19 |
| TR03 | 1 | -- | -- | 1 | 23 | -- | -- | 25 |
| TR04 | 1 | 1 | 1 | 1 | 14 | -- | 3 | 21 |
| TR05 | 1 | -- | -- | 1 | 15 | -- | 1 | 18 |
| TR06 | 1 | -- | 1 | 1 | 20 | -- | 4 | 27 |
| FC01 | -- | -- | -- | 1 | 16 | -- | 2 | 19 |
| FC02 | -- | 1 | -- | 1 | 24 | -- | 2 | 28 |
| FC03 | 1 | 1 | -- | 1 | 28 | -- | 1 | 32 |
| FC04 | -- | -- | -- | 1 | 27 | -- | 3 | 31 |
| FC05 | -- | 1 | -- | 1 | 23 | -- | 2 | 27 |
| DR01 | 1 | 1 | -- | 1 | 16 | -- | -- | 19 |
| DR02 | 1 | 2 | 3 | 2 | 25 | -- | 3 | 36 |
| DR03 | 1 | -- | -- | 1 | 12 | -- | -- | 14 |
| DR04 | -- | 1 | 1 | 1 | 16 | -- | -- | 19 |
| DR05 | 1 | 1 | 1 | 1 | 14 | -- | -- | 18 |
| KM01 | 1 | 1 | 1 | 1 | 19 | -- | 4 | 27 |
| KM02 | -- | 1 | 1 | 1 | 19 | -- | 3 | 25 |
| KM03 | -- | 1 | 1 | 1 | 17 | -- | 6 | 26 |
| KM04 | 1 | 1 | 1 | 1 | 18 | -- | 4 | 26 |
| KM05 | 1 | 2 | 1 | 1 | 15 | -- | 3 | 23 |
| KM06 | -- | 1 | 1 | 1 | 23 | -- | 5 | 31 |
| YH01 | 1 | 3 | 4 | 4 | 9 | -- | 8 | 29 |
| YH02 | 1 | 3 | 6 | 4 | 9 | -- | 12 | 35 |
| YH03 | 1 | 5 | 7 | 5 | 21 | 1 | 24 | 64 |
| YH04 | 1 | 1 | 5 | 3 | 36 | -- | 4 | 50 |
| YH05 | 1 | 1 | 3 | 2 | 9 | -- | 5 | 21 |
| YH06 | 1 | 27 | 6 | 9 | 33 | -- | 12 | 88 |
| ML01 | 1 | 2 | 2 | 3 | 89 | -- | 21 | 118 |
| ML02 | 1 | 4 | 4 | 28 | 86 | 1 | 23 | 147 |
| ML03 | 1 | 12 | 1 | 18 | 94 | -- | 25 | 151 |
| ML04 | 1 | 14 | 1 | 10 | 90 | -- | 26 | 142 |
| ML05 | 1 | 19 | 2 | 21 | 77 | -- | 11 | 131 |
| LX01 | 1 | 2 | 2 | 1 | 15 | -- | 1 | 22 |
| LX02 | 1 | 1 | 1 | 1 | 14 | -- | 1 | 19 |
| LX03 | 1 | 1 | 1 | 1 | 18 | -- | -- | 22 |
| LX04 | 1 | 1 | 1 | 1 | 19 | -- | -- | 23 |
| LX05 | 1 | 1 | 1 | 1 | 8 | -- | 1 | 13 |
| LX06 | 1 | 1 | 1 | 1 | 15 | -- | -- | 19 |
| TY01 | -- | -- | 2 | 4 | 22 | -- | 6 | 34 |
| TY02 | -- | -- | -- | 5 | -- | -- | 6 | 11 |
| TY03 | -- | -- | -- | 3 | -- | -- | 7 | 10 |
| TY04 | -- | -- | -- | 2 | -- | -- | 1 | 3 |
| TY05 | -- | -- | -- | 4 | -- | -- | 6 | 10 |
| PJ01 | 1 | 1 | -- | 1 | 16 | -- | -- | 19 |
| PJ02 | 1 | 1 | -- | 1 | 18 | -- | 1 | 22 |
| PJ03 | 1 | 1 | -- | 1 | 16 | -- | 1 | 20 |
| PJ04 | 1 | 1 | 1 | 1 | 12 | -- | -- | 16 |
| PJ05 | 1 | 1 | 1 | 1 | 14 | -- | -- | 18 |
| ZJ01 | 1 | 1 | 1 | 1 | 15 | -- | 1 | 20 |
| ZJ02 | 1 | 1 | 1 | 1 | 17 | -- | 1 | 22 |
| ZJ03 | 1 | 1 | 1 | 1 | 14 | -- | 1 | 19 |
| ZD01 | 1 | 1 | 1 | 1 | 21 | -- | 3 | 28 |
| ZD02 | 1 | 1 | 1 | 1 | 12 | -- | 2 | 18 |
| YS01 | 1 | 2 | 1 | 1 | 9 | -- | 2 | 16 |
| YS02 | 1 | 2 | 3 | 2 | 2 | -- | 3 | 13 |
| Detection Rate (%) | 75.00 | 78.57 | 67.86 | 100.00 | 92.86 | 3.57 | 80.36 | / |

Note: --: No ASV was detected for this species in this sample.
