# Supplementary material for: DNA metabarcoding unveils authenticity and adulteration in commercial Chinese polyherbal preparations: Renshen Jianpi Wan as a critical case study
Source: Front Pharmacol. 2025 Apr 28;16:1584065. doi: 10.3389/fphar.2025.1584065 (PMC12066679; doi:10.3389/fphar.2025.1584065)
Supplement: Supplementary file 7 [file Table3.docx]

| Supplementary Table 3 Number of ASVs for detected species in RSJPW reference samples using ITS2 and *psb*A-*trn*H markers | | | | | | | | | | | | | | | | |
| --- | --- | --- | --- | --- | --- | --- | --- | --- | --- | --- | --- | --- | --- | --- | --- | --- |
| Herbal ingredients | ITS2 | | | | | | | | *psb*A-*trn*H | | | | | | | |
|  | RF01 | RF02 | RF03 | RF04 | RF05 | RF06 | RF07 | RF08 | RF01 | RF02 | RF03 | RF04 | RF05 | RF06 | RF07 | RF08 |
| Ginseng Radix et Rhizoma (Renshen) | 1 | 1 | 1 | 2 | 2 | 1 | 1 | 1 | 1 | 1 | 1 | 1 | 1 | 1 | 1 | 1 |
| Dioscoreae Rhizoma (Shanyao) | -- | -- | -- | -- | -- | -- | -- | -- | 4 | 3 | 3 | 4 | 3 | 3 | 3 | 4 |
| Aucklandiae Radix (Muxiang) | 3 | 2 | 3 | 4 | 3 | 3 | 3 | 3 | -- | 1 | 1 | -- | -- | -- | -- | 1 |
| Amomi Fructus (Sharen) | 1 | 1 | 3 | 1 | 1 | 1 | 1 | -- | -- | -- | -- | -- | -- | -- | -- | -- |
| Astragali Radix (Huangqi) | 2 | 2 | 2 | 2 | 2 | 2 | 2 | 2 | -- | -- | -- | -- | -- | -- | 1 | 1 |
| Angelicae Sinensis Radix (Danggui) | 6 | 12 | 6 | 5 | 6 | 10 | 8 | 8 | -- | -- | -- | -- | -- | -- | -- | -- |
| Ziziphi Spinosae Semen (Suanzaoren) | 3 | 2 | 3 | 4 | 4 | 3 | 3 | 3 | 12 | 11 | 12 | 17 | 9 | 17 | 8 | 11 |
| Polygalae Radix (Yuanzhi) | 1 | 1 | 1 | 1 | 1 | 1 | 1 | 1 | -- | -- | -- | -- | -- | -- | -- | -- |
| **Panacis Quinquefolii Radix (Xiyangshen)** | 1 | 1 | 1 | 1 | -- | -- | -- | -- | -- | -- | -- | -- | -- | -- | -- | -- |

Note:--: No ASV was detected for this species in this sample.
