# Supplementary material for: DNA metabarcoding unveils authenticity and adulteration in commercial Chinese polyherbal preparations: Renshen Jianpi Wan as a critical case study
Source: Front Pharmacol. 2025 Apr 28;16:1584065. doi: 10.3389/fphar.2025.1584065 (PMC12066679; doi:10.3389/fphar.2025.1584065)
Supplement: Supplementary file 8 [file Table4.docx]

| Supplementary Table 4 Number of ASVs for detected species in commercial RSJPW samples based on ITS2 sequences | | | | | | | | | | | |
| --- | --- | --- | --- | --- | --- | --- | --- | --- | --- | --- | --- |
| Ingredient  Batch code | Ginseng Radix et Rhizoma | Atractylodis Macrocephalae Rhizoma | Citri Reticulatae Pericarpium | Aucklandiae Radix | Amomi Fructus | Astragali Radix | Angelicae Sinensis Radix | Ziziphi Spinosae Semen | Polygalae Radix | Other species | Total |
| TR01 | 1 | 1 | -- | 1 | 1 | 2 | 1 | 8 | -- | 3 | 18 |
| TR02 | 1 | -- | -- | 1 | 1 | 2 | 1 | 9 | -- | 1 | 16 |
| TR03 | 1 | -- | -- | 1 | 1 | 2 | 1 | 11 | -- | 4 | 21 |
| TR04 | 1 | -- | -- | 1 | 1 | 2 | 2 | 10 | -- | 4 | 21 |
| TR05 | 1 | -- | -- | 1 | 1 | 2 | 3 | 12 | -- | 4 | 24 |
| TR06 | 1 | -- | -- | 1 | 2 | 3 | 1 | 7 | 1 | 8 | 24 |
| FC01 | -- | -- | -- | -- | 3 | 1 | 1 | 10 | -- | 5 | 20 |
| FC02 | 1 | -- | -- | -- | 4 | 1 | -- | 8 | -- | 5 | 19 |
| FC03 | 1 | -- | -- | -- | 3 | 1 | 1 | 9 | -- | 1 | 16 |
| FC04 | -- | -- | 1 | -- | 5 | 1 | 1 | 5 | -- | 8 | 21 |
| FC05 | 1 | -- | -- | 1 | 2 | 1 | 1 | 7 | -- | 7 | 20 |
| DR01 | 1 | 1 | 1 | -- | 1 | 2 | 1 | 8 | 1 | 3 | 19 |
| DR02 | 1 | 1 | -- | -- | 4 | 1 | 1 | 6 | -- | 13 | 27 |
| DR03 | 1 | 1 | 1 | -- | 2 | 2 | 1 | 8 | 1 | 1 | 18 |
| DR04 | 1 | -- | -- | -- | -- | 2 | 1 | 5 | 1 | 3 | 13 |
| DR05 | 1 | -- | 1 | -- | 1 | 2 | 1 | 4 | 1 | 2 | 13 |
| KM01 | 1 | 1 | -- | 3 | 1 | 3 | 1 | 5 | 1 | 4 | 20 |
| KM02 | 1 | 1 | 1 | 4 | 1 | 3 | 1 | 6 | 1 | 17 | 36 |
| KM03 | 1 | 1 | -- | 3 | -- | 3 | 1 | 4 | 1 | 9 | 23 |
| KM04 | 1 | 1 | 1 | 2 | -- | 3 | 1 | 5 | 1 | 3 | 18 |
| KM05 | 1 | -- | -- | 1 | 5 | 2 | 1 | 6 | 1 | 7 | 24 |
| KM06 | 1 | 1 | -- | 3 | 1 | 4 | 1 | 6 | 1 | 18 | 36 |
| YH01 | 1 | -- | -- | 1 | 1 | 3 | 2 | 4 |  | 18 | 30 |
| YH02 | 1 | -- | -- | 1 | 2 | 3 | 1 | 3 | 1 | 19 | 31 |
| YH03 | 1 | -- | -- | 1 | 3 | 3 | 1 | 3 | 1 | 32 | 45 |
| YH04 | 1 | -- | -- | 1 | 1 | 4 | 1 | 4 | -- | 15 | 27 |
| YH05 | 1 | -- | -- | 2 | 1 | 3 | 2 | 4 | -- | 16 | 29 |
| YH06 | 1 | 1 | 1 | 3 | 2 | 4 | 1 | 4 | -- | 28 | 45 |
| ML01 | 1 | -- | -- | -- | 4 | -- | -- | 9 | -- | 4 | 18 |
| ML02 | 1 | 1 | -- | -- | 4 | 1 | -- | 8 | -- | 13 | 28 |
| ML03 | 1 | 2 | -- | 1 | 5 | 1 | 1 | 10 | -- | 25 | 46 |
| ML04 | 1 | -- | -- | 1 | 5 | 1 | -- | 9 | -- | 18 | 35 |
| ML05 | 1 | -- | -- | -- | 6 | 1 | -- | 9 | -- | 10 | 27 |
| LX01 | 1 | 1 | -- | 1 | 1 | 3 | 1 | 6 | 1 | 1 | 16 |
| LX02 | 1 | 1 | -- | 1 | -- | 3 | 2 | 4 | 1 | -- | 13 |
| LX03 | 1 | -- | -- | 1 | 1 | 3 | 1 | 7 | 1 | 1 | 16 |
| LX04 | 1 | -- | -- | -- | 2 | 2 | 1 | 9 | 1 | 1 | 17 |
| LX05 | 1 | 1 | -- | 1 | 1 | 3 | 1 | 5 | -- | -- | 13 |
| LX06 | 1 | -- | 1 | 1 | -- | 3 | 1 | 5 | 1 | 2 | 15 |
| TY01 | 1 | -- | -- | -- | 7 | 1 | 3 | 58 | -- | 74 | 144 |
| TY02 | 1 | -- | -- | -- | 18 | 1 | 2 | 82 | -- | 71 | 175 |
| TY03 | 1 | 1 | 1 | -- | 9 | 1 | 1 | 57 | -- | 65 | 136 |
| TY04 | 1 | 3 | -- | -- | 10 | 2 | 2 | 61 | -- | 75 | 154 |
| TY05 | -- | 1 | 1 | -- | 17 | 1 | 1 | 52 | 1 | 58 | 132 |
| PJ01 | 1 | -- | -- | -- | 3 | -- | 1 | 10 | -- | 2 | 17 |
| PJ02 | 1 | -- | -- | -- | 1 | -- | 1 | 8 | -- | 5 | 16 |
| PJ03 | 1 | 1 | -- | -- | 2 | 2 | 1 | 8 | -- | 4 | 19 |
| PJ04 | 1 | 1 | -- | -- | 1 | 4 | 1 | 9 | -- | 5 | 22 |
| PJ05 | 1 | -- | 1 | -- | 2 | 2 | 1 | 7 | -- | 5 | 19 |
| ZJ01 | 1 | -- | -- | 1 | -- | 3 | 1 | 7 | 1 | 1 | 15 |
| ZJ02 | 2 | -- | -- | 1 | -- | 3 | 1 | 8 | 1 | -- | 16 |
| ZJ03 | 1 | -- | -- | 1 | -- | 3 | 1 | 7 | 1 | 2 | 16 |
| ZD01 | 1 | -- | 1 | 1 | 1 | 3 | 1 | 8 | 1 | 11 | 28 |
| ZD02 | 1 | -- | 1 | 1 | 1 | 4 | 1 | 9 | 1 | 17 | 36 |
| YS01 | 1 | 1 | 1 | 2 | 1 | 3 | 1 | 5 | 1 | 5 | 21 |
| YS02 | 1 | -- | 2 | 1 | 1 | 4 | 1 | 3 | 2 | 4 | 19 |
| Detection Rate (%) | 94.64 | 37.5 | 26.79 | 58.93 | 85.71 | 94.64 | 91.07 | 100.00 | 46.43 | 94.64 | / |

Note: --: No ASV was detected for this species in this sample.
