# Supplementary material for: DNA metabarcoding unveils authenticity and adulteration in commercial Chinese polyherbal preparations: Renshen Jianpi Wan as a critical case study
Source: Front Pharmacol. 2025 Apr 28;16:1584065. doi: 10.3389/fphar.2025.1584065 (PMC12066679; doi:10.3389/fphar.2025.1584065)
Supplement: Supplementary file 9 [file Table6.docx]

| Supplementary Table 6 Number of ASV reads for detected species in commercial RSJPW samples based on ITS2 sequences | | | | | | | | | |
| --- | --- | --- | --- | --- | --- | --- | --- | --- | --- |
| Ingredient  Batch code | Ginseng Radix et Rhizoma | Atractylodis Macrocephalae Rhizoma | Citri Reticulatae Pericarpium | Aucklandiae Radix | Amomi Fructus | Astragali Radix | Angelicae Sinensis Radix | Ziziphi Spinosae Semen | Polygalae Radix |
| TR01 | 207 | 9 | -- | 56 | 39 | 718 | 403 | 31,700 | -- |
| TR02 | 553 | -- | -- | 99 | 34 | 1,439 | 404 | 29,975 | -- |
| TR03 | 175 | -- | -- | 20 | 29 | 296 | 157 | 31,241 | -- |
| TR04 | 294 | -- | -- | 89 | 210 | 1,586 | 1,102 | 29,071 | -- |
| TR05 | 560 | -- | -- | 32 | 29 | 1,313 | 5,043 | 27,624 | -- |
| TR06 | 1,011 | -- | -- | 84 | 298 | 3,195 | 4,274 | 21,491 | 12 |
| FC01 | -- | -- | -- | -- | 218 | 19 | 167 | 19,832 | -- |
| FC02 | 61 | -- | -- | -- | 580 | 18 | -- | 20,984 | -- |
| FC03 | 17 | -- | -- | -- | 257 | 10 | 8 | 21,123 | -- |
| FC04 | -- | -- | 8 | -- | 744 | 58 | 25 | 19,098 | -- |
| FC05 | 31 | -- | -- | 9 | 155 | 34 | 17 | 21,103 | -- |
| DR01 | 282 | 49 | 19 | -- | 25 | 876 | 224 | 19,605 | 19 |
| DR02 | 18 | 116 | -- | -- | 5,564 | 154 | 222 | 11,591 | -- |
| DR03 | 461 | 17 | 3 | -- | 159 | 1,020 | 786 | 18,155 | 64 |
| DR04 | 144 | -- | -- | -- | -- | 1,386 | 3,984 | 12,023 | 51 |
| DR05 | 780 | -- | 13 | -- | 68 | 1,841 | 4,908 | 7,980 | 82 |
| KM01 | 62 | 21 | -- | 350 | 10 | 8,357 | 60 | 7,312 | 46 |
| KM02 | 157 | 35 | 12 | 795 | 21 | 8,208 | 116 | 7,773 | 108 |
| KM03 | 41 | 41 | -- | 399 | -- | 7,319 | 96 | 7,747 | 25 |
| KM04 | 93 | 26 | 5 | 382 | -- | 7,604 | 57 | 7,785 | 146 |
| KM05 | 10 | -- | -- | 52 | 760 | 3,289 | 383 | 12,370 | 36 |
| KM06 | 70 | 45 | -- | 498 | 23 | 6,474 | 120 | 10,574 | 36 |
| YH01 | 1,323 | -- | -- | 32 | 40 | 11,617 | 3433 | 1,376 | -- |
| YH02 | 1,328 | -- | -- | 26 | 26 | 11,611 | 1,949 | 1,491 | 11 |
| YH03 | 66 | -- | -- | 28 | 211 | 1,799 | 91 | 979 | 21 |
| YH04 | 1,422 | -- | -- | 24 | 58 | 11,970 | 3,360 | 4,905 | -- |
| YH05 | 1,336 | -- | -- | 125 | 30 | 7,485 | 3,050 | 6,384 | -- |
| YH06 | 518 | 21 | 29 | 85 | 84 | 11,700 | 2,954 | 6,779 | -- |
| ML01 | 15 | -- | -- | -- | 1,238 | -- | -- | 18,169 | -- |
| ML02 | 20 | 73 | -- | -- | 1,175 | 9 | -- | 18,821 | -- |
| ML03 | 38 | 226 | -- | 9 | 1,379 | 19 | 25 | 21,917 | -- |
| ML04 | 28 | -- | -- | 10 | 1,564 | 28 | -- | 24,995 | -- |
| ML05 | 42 | -- | -- | -- | 1,453 | 22 | -- | 23,624 | -- |
| LX01 | 478 | 12 | -- | 25 | 20 | 7,012 | 4,601 | 7,758 | 57 |
| LX02 | 627 | 10 | -- | 25 | -- | 6,529 | 4,592 | 7,393 | 44 |
| LX03 | 263 | -- | -- | 32 | 16 | 3,644 | 1,723 | 15,213 | 25 |
| LX04 | 212 | -- | -- | -- | 277 | 2,111 | 568 | 19,854 | 12 |
| LX05 | 303 | 3 | -- | 33 | 62 | 3,966 | 3,951 | 12,685 | -- |
| LX06 | 583 | -- | 27 | 43 | -- | 5,430 | 5,036 | 8,758 | 135 |
| TY01 | 55 | -- | -- | -- | 361 | 110 | 214 | 8,320 | -- |
| TY02 | 13 | -- | -- | -- | 1,430 | 146 | 366 | 9,917 | -- |
| TY03 | 34 | 21 | 74 | -- | 443 | 178 | 89 | 5,697 | -- |
| TY04 | 14 | 100 | -- | -- | 580 | 216 | 326 | 6,392 | -- |
| TY05 | -- | 13 | 76 | -- | 757 | 91 | 91 | 4,283 | 53 |
| PJ01 | 124 | -- | -- | -- | 1,470 | -- | 435 | 22,215 | -- |
| PJ02 | 255 | -- | -- | -- | 84 | -- | 890 | 25,251 | -- |
| PJ03 | 302 | 26 | -- | -- | 182 | 2,585 | 380 | 20,988 | -- |
| PJ04 | 237 | 15 | -- | -- | 25 | 3,152 | 1,761 | 19,093 | -- |
| PJ05 | 133 | -- | 5 | -- | 216 | 1,174 | 1,749 | 19,584 | -- |
| ZJ01 | 166 | -- | -- | 10 | -- | 1,251 | 1,689 | 20,334 | 26 |
| ZJ02 | 414 | -- | -- | 21 | -- | 4,141 | 2,009 | 14,281 | 70 |
| ZJ03 | 398 | -- | -- | 28 | -- | 4,700 | 2,113 | 12,384 | 162 |
| ZD01 | 360 | -- | 53 | 36 | 15 | 4,127 | 1,933 | 16,014 | 159 |
| ZD02 | 321 | -- | 21 | 46 | 29 | 4,442 | 1,995 | 14,808 | 133 |
| YS01 | 1,073 | 47 | 60 | 113 | 230 | 6,800 | 2,612 | 12,016 | 341 |
| YS02 | 892 | -- | 178 | 74 | 35 | 19,131 | 1,339 | 784 | 478 |
| Total | 18,420 | 926 | 583 | 3,690 | 22,713 | 192,410 | 77,880 | 829,619 | 2,352 |
| Relative  abundance (%) | 1.46 | 0.07 | 0.05 | 0.29 | 1.80 | 15.23 | 6.16 | 65.67 | 0.19 |

Note: --: No reads was detected for this species in this sample; Relative abundance = (Number of ASV reads for the species / Total number of reads) × 100%
